# Glutamate and GABA_A_ receptor crosstalk mediates homeostatic regulation of neuronal excitation in the mammalian brain

**DOI:** 10.1038/s41392-022-01148-y

**Published:** 2022-10-03

**Authors:** Ya Wen, Zhifang Dong, Jun Liu, Peter Axerio-Cilies, Yehong Du, Junjie Li, Long Chen, Lu Zhang, Lidong Liu, Jie Lu, Ning Zhou, Dong Chuan Wu, Yu Tian Wang

**Affiliations:** 1 DM Centre for Brain Health and Department of Medicine, Vancouver Coastal Health Research Institute, University of British Columbia, Vancouver, BC V6T 2B5 Canada; 2 Pediatric Research Institute, Ministry of Education Key Laboratory of Child Development and Disorders, National Clinical Research Center for Child Health and Disorders, China International Science and Technology Cooperation Base of Child Development and Critical Disorders, Chongqing Key Laboratory of Translational Medical Research in Cognitive Development and Learning and Memory Disorders, Children’s Hospital of Chongqing Medical University, Chongqing, 400014 P.R. China; 3 iHuman Institue, ShanghaiTech University, Shanghai, P.R. China

**Keywords:** Cellular neuroscience, Molecular neuroscience

## Abstract

Maintaining a proper balance between the glutamate receptor-mediated neuronal excitation and the A type of GABA receptor (GABA_A_R) mediated inhibition is essential for brain functioning; and its imbalance contributes to the pathogenesis of many brain disorders including neurodegenerative diseases and mental illnesses. Here we identify a novel glutamate-GABA_A_R interaction mediated by a direct glutamate binding of the GABA_A_R. In HEK293 cells overexpressing recombinant GABA_A_Rs, glutamate and its analog ligands, while producing no current on their own, potentiate GABA-evoked currents. This potentiation is mediated by a direct binding at a novel glutamate binding pocket located at the α^+^/β^−^ subunit interface of the GABA_A_R. Moreover, the potentiation does not require the presence of a γ subunit, and in fact, the presence of γ subunit significantly reduces the potency of the glutamate potentiation. In addition, the glutamate-mediated allosteric potentiation occurs on native GABA_A_Rs in rat neurons maintained in culture, as evidenced by the potentiation of GABA_A_R-mediated inhibitory postsynaptic currents and tonic currents. Most importantly, we found that genetic impairment of this glutamate potentiation in knock-in mice resulted in phenotypes of increased neuronal excitability, including decreased thresholds to noxious stimuli and increased seizure susceptibility. These results demonstrate a novel cross-talk between excitatory transmitter glutamate and inhibitory GABA_A_R. Such a rapid and short feedback loop between the two principal excitatory and inhibitory neurotransmission systems may play a critical homeostatic role in fine-tuning the excitation-inhibition balance (E/I balance), thereby maintaining neuronal excitability in the mammalian brain under both physiological and pathological conditions.

## Introduction

Neuronal excitability is primarily controlled by a balance between synaptic excitation and inhibition. In the mammalian brain, synaptic excitation is predominantly mediated by the excitatory transmitter glutamate acting on ionotropic glutamate receptor-gated cationic channels; while synaptic inhibition is primarily mediated by the inhibitory transmitter γ-aminobutyric acid (GABA) acting on the ionotropic A type GABA receptor (GABA_A_R). Mechanisms maintaining the normal level of glutamate-mediated synaptic excitation and GABA-mediated synaptic inhibition are fundamentally important for physiological brain functions, including learning and memory. Dysfunction of those mechanisms causes the excitation-inhibition imbalance, leading to the pathogenesis of various neuropathological disorders from acute neuronal network dysfunction, such as epilepsy, and neurodegeneration, including cerebral ischemia and Alzheimer’s disease, to cognitive disorders, including schizophrenia and autism.^1–4^ Thus, further study of the mechanisms that regulate the neuronal excitation-inhibition balance may significantly contribute to our current understanding of brain physiology and pathophysiology.

The interaction between excitation and inhibition occurs at the circuit, cellular, and even molecular levels. At the circuit level, E/I balance is required for both long-range connectivity between neural structures and local connectivity within a brain region. Pyramidal neurons can activate excitatory pyramidal neurons or inhibitory interneurons to enhance or inhibit the excitatory output through feedforward or feedback mechanisms.^5^ At the cellular level, E/I balance can be regulated between excitatory and inhibitory synapses onto the same neuron. Activation of GABA_A_Rs located at dendritic shafts reduces the excitatory synaptic input to adjacent dendritic spines to confine the propagation of excitatory postsynaptic potentials in a rapid and instant mode. Activation of metabotropic glutamate or GABA receptors leads to indirect and long-lasting effects on neuronal excitability. For example, GABA can activate G protein-coupled GABA_B_ receptors at the excitatory presynaptic terminals to inhibit glutamate release by decreasing calcium channel conductance or by enhancing potassium channel activity to hyperpolarize the cell membrane.^6^ In a similar pattern, glutamate can activate metabotropic glutamate receptors to modulate GABA_A_R activity and regulate inhibitory synaptic transmission.^7^ All these interactions are based on the binding specificity between the neurotransmitter (glutamate or GABA) and their receptors (glutamate receptors or GABA_A_Rs, respectively). It is rarely reported that the excitatory neurotransmitter directly interacts with inhibitory receptors or vice versa. Interestingly, Johnson and Ascher have found that inhibitory transmitter glycine is required for activation of the N-methyl-D-aspartate receptor (NMDAR), which is a subtype of glutamate receptors, and later proved that glycine directly binds to and allosterically modulates NMDARs.^8^ Stelzer and Wong reported that glutamate potentiated GABA_A_R-mediated responses in acutely isolated hippocampal neurons.^9^ Our previous studies also revealed that glutamate potentiates glycine receptors (GlyR), which is the major inhibitory receptor in the brain stem and spinal cord.^10^ These studies indicate that in certain circumstances, the excitatory neurotransmitter glutamate may directly affect inhibitory transmission. However, compared to the high sensitivity of NMDARs to glycine potentiation that requires only nano- to low-micromolar levels of glycine, the glutamate concentration for modulating GlyR- or GABA_A_R-mediated responses needs to reach micro- to millimolar levels. Considering the mechanisms of synaptic clearance, the higher concentration of glutamate cannot be reached at inhibitory postsynaptic GlyR or GABA_A_Rs by diffusion. Nevertheless, recent studies have demonstrated that the inhibitory pre-synapses in different brain regions, including hippocampus, habenula, and ventral tegmental area,^11–15^ can co-release glutamate and GABA onto the same postsynaptic site, which may allow high concentration of glutamate to reach GABA_A_Rs, implying a link between glutamate modulation of GABA_A_R and physiological consequences. Hence, it is important to understand whether glutamate potentiation on GABA_A_R is mediated by direct interaction or by indirectly mechanisms.

GABA_A_Rs are heteropentameric receptor ion channels, assembled by combining homologous subunits from different classes (1−6), β (1−3), γ (1−3), δ, ε, π, ρ (1−3), and θ^2^. The majority of synaptic GABA_A_Rs pentamer is composed by two α, two β, and one γ. Among them, α1, β2/3, and γ2 combination is one of the most ubiquitous form of GABA_A_R in the brain. Different compositions of GABA_A_Rs play distinct roles in physiological or pathophysiological conditions, such as the involvement of the α1 subunit in sedation and the α2 subunit in anxiety.^16–18^ Being a central role in inhibitory processes, the GABA_A_R is a vital drug target for treatments of numerous neurobiological diseases and underlines allosteric modulation by a variety of endogenous molecules and exogenous substrates, including steroids, alcohol, benzodiazepine (BZ), volatile anaesthetics and general anaesthetics.^19^ The benzodiazepine group of drugs are a typical example of GABA_A_R allosteric modulator with extensive clinical usage. These allosteric modulators target on different conformational regions of GABA_A_Rs. For example, the GABA binding site is located at the interface between the α and β subunits (β+/α− interface), while the benzodiazepine binding site is located at the α and γ subunits interface (α+/γ− interface).^19^ Recently, Ramerstorfer et al. found that the β and α subunits interface (α+/β− interface) may be another potential allosteric modulation site.^20^ Therefore, if glutamate directly binds to and potentiates GABA_A_R functions via allosteric modulation, discovery of this binding site may provide future drug targets for GABA_A_R-associated brain diseases.

In the present study, we identify a novel allosteric glutamate-binding site on the GABA_A_R, at which glutamate and many of its analogs can allosterically potentiate the receptor function. This previously unrecognized novel cross-talk between the two classic neurotransmitter systems blurs the traditional distinction between excitatory and inhibitory transmitters, and promotes us to further investigate its physiological and/or pathological roles. Using a genetic elimination of this novel glutamate modulation, we reveal that it may function as an essential homeostatic feedback mechanism in controlling excitation-inhibition balance, and hence maintaining a normal level of neuronal excitability in the mammalian brain.

## Result

### Glutamate and its analogs bind to and exert a positive allosteric modulation of GABA_A_Rs

**Fig. 1:**
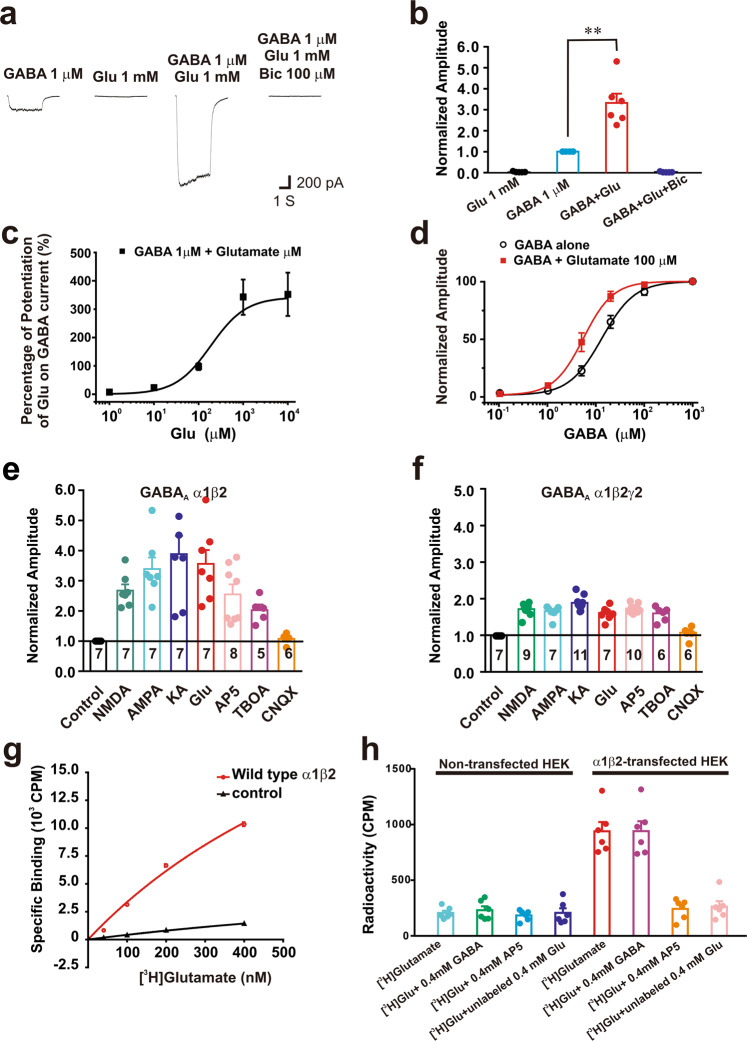
Glutamate-like ligands potentiate GABA_A_R-mediated currents via a direct binding to the receptor in HEK293 cells. HEK293 cells were transiently transfected with rat GABA_A_R α1/β2 (**a**–**e**) or α1/β2/γ2 (**f**) subunits. Whole-cell voltage-clamp recordings were performed with a chloride-based intracellular recording solution at a holding membrane potential of −60 mV. GABA_A_R-mediated currents were evoked by perfusion of GABA alone and/or in combination with a glutamate-like ligand through a computer-driven multi-barrel fast perfusion system. **a** Representative traces showing that glutamate potentiated GABA_A_R-mediated currents. Glutamate (1 mM) produced no detectable currents on its own, but potentiated GABA currents; these currents were blocked by bicuculline (100 μM). **b** Bar graph summarizing glutamate potentiation of GABA_A_R mediated currents from six individual cells shown in **a**. **c** Dose-response curve showing glutamate–induced potentiation of the currents evoked by 1 μM GABA (*n* = 7). **d** GABA dose-response curves constructed from currents recorded in the presence and absence of 100 μM glutamate showing the glutamate-induced left shifting of GABA dose-response curve. **e**, **f** Bar graphs summarizing the potentiation of GABA (1 μM)-induced currents by glutamate or its analogs in HEK293 cells expressing α1/β2 (**e**) or α1/β2/γ2 (**f**) GABA_A_Rs (Numbers in each bars in **e** and **f** indicate the number of independent recording in each groups). **g**, **h** Glutamate binds to the α1/β2 GABA_A_Rs via a site that is not overlapped with the GABA binding site. [^3^H]glutamate binding assays showed glutamate specifically bound to the plasma membranes of HEK293 transiently transfected with α1β2 GABA_A_Rs, but not that of non-transfected HEK293 controls (**g**; *n* = 3). The specific [^3^H]glutamate (40 nM) binding was competitively blocked by a high concentration of non-radiolabeled glutamate or AP5, but not affected by a high concentration of non-radiolabeled GABA (**h**; *n* = 6)

To determine if glutamate can indeed potentiate GABA_A_R responses and if so, to characterize the detailed underlying mechanisms, we first investigated if glutamate or its analogs have any effect on GABA-evoked responses in HEK293 cells that transiently expressed recombinant rat GABA_A_Rs, but not any known glutamate receptors. Co-expression of the α and β subunits is the minimum requirement for a functional recombinant GABA_A_R expressed in a heterologous cell line such as HEK293, while co-expression of the α, β and γ subunits is required for the recombinant receptor with a full pharmacological profile.^2^ We therefore transiently expressed either rat α1β2 or α1β2γ2 subunits in HEK293 cells. Whole-cell patch clamp recordings of GABA evoked currents with chloride-based pipette solutions were performed under voltage clamp at a holding potential of −60mV. Consistent with the expression of functional recombinant GABA_A_Rs, fast perfusion of GABA (1 μM) induced inward currents in HEK293 cells expressing α1β2 (Fig. 1a). Perfusion of glutamate (1 mM) to the same cells produced no noticeable current on its own (Supplementary Fig. S5), confirming the lack of ionotropic glutamate receptors in these cells (Fig. 1a, b). However, when glutamate was co-applied with GABA, it resulted in a more than 3-fold increase in the amplitude of GABA-induced currents (Fig. 1b; 331.2 ± 44.5%, *n* = 6, *p* < 0.001). Both basal and glutamate-increased GABA currents were blocked by the GABA_A_R antagonist bicuculline (Fig. 1a, b; Bic, 100 μM), demonstrating that these currents, both in the absence and presence of glutamate, are entirely gated through the Cl- channel of the GABA_A_R. Dose-response analysis of the glutamate potentiation on 1 μM GABA-induced currents indicates that the EC_50_ of glutamate potentiation was close to 180 μM, with the lowest effective dose (20% potentiation) being around 30 μM (Fig. 1c). At a fixed glutamate concentration of 100 μM, glutamate produced a leftward shift in the GABA dose-response curve, reducing the EC_50_ of GABA from 13.19 ± 1.08 μM to 5.46 ± 1.10 μM. This reduction of EC_50_ was not associated with an obvious alteration in the Hill coefficient (1.28 ± 0.12 and 1.42 ± 0.18 in the absence and presence of 100 µM glutamate, respectively), indicating that glutamate may affect GABA binding affinity on GABA_A_Rs (Fig. 1d). Notably, glutamate had a greater potentiation effect on currents induced by low-doses rather than high-doses of GABA, and exhibited almost no potentiation effect when GABA reached a saturated concentration (Fig. 1d).

The amino and α-carboxyl groups of glutamate molecules are known to be required for glutamate binding to various known glutamate-binding proteins/receptors, and are also present in many glutamate analogs.^21–25^ We next tested if glutamate analogs containing these groups might be capable of mimicking glutamate to potentiate GABA_A_R currents. We found that AMPA (100 μM; an agonist for AMPA type glutamate receptor), kainic acid (100 μM; an agonist for both kainate and AMPA glutamate receptors), and NMDA (100 μM; an agonist for NMDA receptor) all mimicked glutamate and enhanced GABA-mediated currents in HEK293 cells expressing α1β2 GABA_A_Rs (Fig. 1e; AMPA, 339.2 ± 38.0%, *n* = 7; *p* < 0.001; kainic acid, 388.2 ± 61.0%, *n* = 7; *p* < 0.01; and NMDA, 267.6 ± %, *n* = 7; *p* < 0.001; compared with currents induced by GABA alone). Moreover, AP5 (100 μM, a competitive antagonist for NMDARs) and TBOA (100 μM, a competitive antagonist for glutamate transporters) also greatly potentiated GABA evoked currents (Fig. 1e; AP5, 254.9 ± 33.0%, *n* = 8; *p* < 0.001; TBOA, 202.8 ± 18.3%, *n* = 5; *p* < 0.01). In contrast, CNQX (10 μM; a competitive antagonist for non-NMDA receptor) did not potentiate GABA evoked currents (Fig. 1e; CNQX, 107.3 ± 6.5 %, *n* = 6; *p* > 0.05). Increasing the concentration of CNQX to 1 mM still did not affect GABA-evoked currents (data not shown). We further found that the non-competitive NMDA receptor antagonist MK-801, which does not have the amino and the α-carboxyl groups of glutamate and acts at a site on the receptor different from glutamate binding sites, did not affect GABA currents in the α1β2 receptors-expressing cells (data not shown). These data indicate the selectivity of this site for glutamate-related structure.

As shown in Fig. 1f, glutamate and its analogs (100 μM each) also potentiated GABA currents in HEK293 cells expressing the recombinant α1β2γ2 subunits (Fig. 1f; AMPA: 165.8 ± 6.2%, *n* = 7; *p* < 0.01; kainic acid: 188.7 ± 5.5%, *n* = 11; *p* < 0.001; NMDA: 171.7 ± 5.4%, *n* = 9; *p* < 0.01; AP5: 173.3 ± 3.7%, *n* = 10; *p* < 0.001; glutamate: 169.3 ± 6.9%, *n* = 7; *p* < 0.05; and TBOA: 159.9 ± 7.8%, *n* = 6; *p* < 0.05). Interestingly, the level of the potentiation appears to be much smaller than that observed in the α1β2 expressing cells (Fig. 1e). Also, CNQX had little effect on GABA currents (Fig. 1f; CNQX: 105.6 ± 6.5%, *n* = 6; *p* > 0.05). Together, these results strongly suggest that glutamate can allosterically potentiate the function of GABA_A_Rs. Moreover, this glutamate modulation does not require the presence of a γ subunit. In fact, the γ subunit may negatively impact the glutamate potentiation.

To determine if the allosteric modulation of GABA_A_Rs by glutamate is a result of direct binding of glutamate to the receptor itself, we performed [^3^H]glutamate binding assays using plasma membranes isolated from HEK293 cells overexpressing the α1 and β2 subunits, with the plasma membranes from the non-transfected HEK293 cells as the controls. As shown in Fig. 1g, h, we found that compared with membranes isolated from the non-transfected cells, there was a dose-dependent [^3^H]glutamate binding to membranes of GABA_A_R overexpressing cells (Fig. 1g). To determine if the binding is specific for glutamate and its analogs, and to determine the relationship between the glutamate binding site and the GABA binding site on the receptor, we next performed competition assays with high concentrations (0.4 mM) of cold glutamate, AP5 or GABA. We found that both glutamate and AP5 could efficiently compete for [^3^H]glutamate binding, reducing the binding activity in the α1β2 expressing cells to a level similar to the nonspecific background activity observed in the non-transfected cells (Fig. 1h; non-transfected control: 204.0 ± 20.6 CPM, *n* = 6; [^3^H]glutamate only: 938.2 ± 82.7 CPM, *n* = 6; *p* < 0.05 of control; [^3^H]glutamate + non-radiolabeled glutamate: 262.2 ± 47.8 CPM, *n* = 6; *p* > 0.05 of control; [^3^H]glutamate + AP5: 240.8 ± 36.8 CPM, *n* = 6; *p* > 0.05 of control). In contrary, GABA failed to alter the [^3^H]glutamate binding activity (Fig. 1h; [^3^H]glutamate + GABA: 939.2 ± 88.8 CPM, *n* = 6; *p* < 0.05 of control). These results indicate that the glutamate-binding site represents a previously unrecognized site that does not overlap with the known GABA-binding site. We named this novel glutamate binding site as glutamate site on the GABA_A_R.

### Activation of glutamate-binding sites on the native GABA_A_R potentiates GABA_A_R-mediated currents in cultured hippocampal neurons

**Fig. 2:**
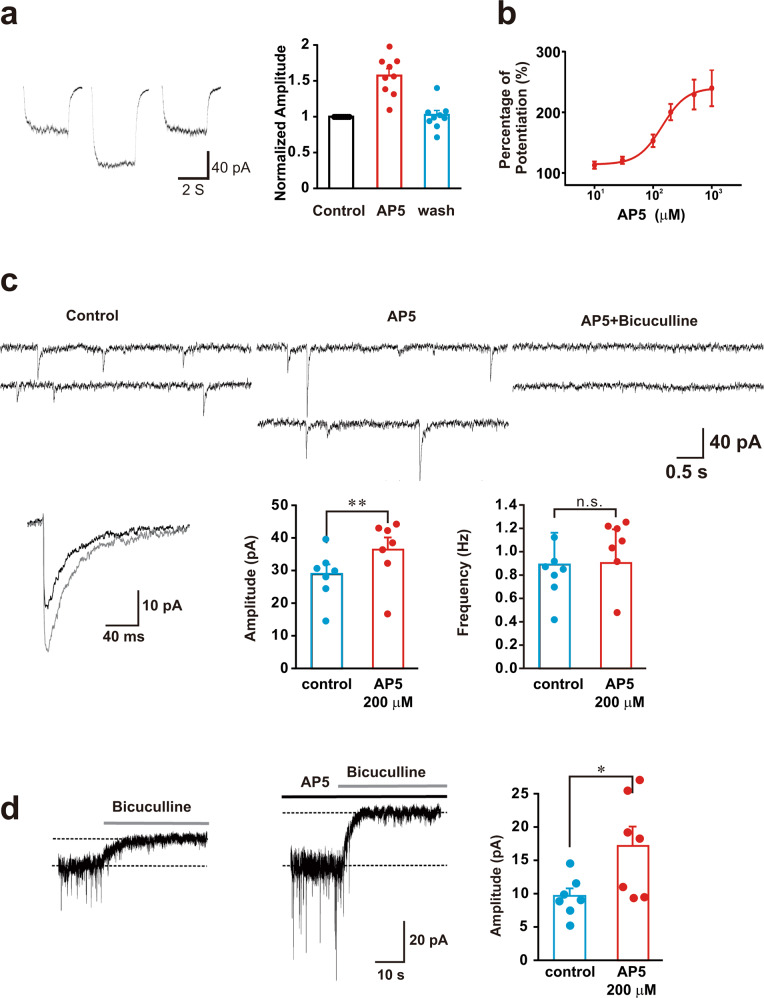
AP5 potentiates the GABA_A_R-mediated currents in cultured hippocampal neurons. Whole-cell currents were recorded with a Cl-based intracellular recording solution under voltage-clamp configuration at a holding membrane potential of −60mV. **a** AP5 reversibly potentiates GABA-induced currents. Left: Representative current traces showed GABA currents induced by fast perfusion of GABA (0.5 μM) before, during, and washout of AP5 (100 μM). Quantified results from nine neurons were shown in the right panel. **b** Dose-response relationship of AP5 potentiation of GABA (0.5 μM) currents (*n* = 6). **c** AP5 potentiates miniature inhibitory postsynaptic currents (mIPSCs) in the presence of 0.5 μM TTX and 10 μM CNQX. AP5 (200 μM; AP5) increased the amplitude, but not the frequency, of mIPSCs. mIPSCs in the presence of AP5 were blocked by bicuculline (10 μM; AP5 + Bicuculline). Averaged traces of mIPSC events before (black) and after AP5 treatment (gray) from the same neurons were shown in the bottom left panel (*n* = 7). **d** AP5 potentiates GABA_A_R-mediated tonic currents (the upward shifting of the baseline) revealed by the perfusion of bicuculline (10 μM) in presence or absence of AP5 (200 µM). Representative traces taken before and after AP5 application were shown in left and middle panels, respectively, and group data from seven individual neurons individual neurons were quantified in the bar graph on the right

Results shown above demonstrate that AP5 can mimic glutamate and allosterically potentiate the function of GABA_A_R by binding directly to and activating the glutamate sites of the GABA_A_R. As an NMDA receptor antagonist, AP5 does not activate any known native ionotropic glutamate receptors, and would be an ideal glutamate analog for characterizing glutamate modulation of native GABA_A_Rs in neurons. We therefore used AP5 as a glutamate analog on this novel glutamate allosteric modulation of function of native GABA_A_Rs in cultured hippocampal neurons. As shown in Fig. 2a, fast perfusion of GABA at a 30 s interval reliably evoked inward GABA currents. Bath application of AP5 (100 μM) reversibly increased the amplitude of GABA currents (Fig. 2a; 157.7 ± 9.1% of the control, *n* = 9; *p* < 0.01). The dose-response analysis showed that AP5 dose-dependently enhanced the GABA currents with an EC_50_ of 150 ± 27 μM (Fig. 2b). As the currents were recorded in the presence of TTX, this potentiation was likely a direct effect of AP5 on the neurons under recording. To further rule out the possibility that this potentiation could be due to an indirect effect of AP5 in blocking ionotropic native NMDA receptors, we first blocked ionotropic NMDA receptors with the MK801, a NMDA receptor open-channel blocker that cannot bind to and potentiate GABA_A_R function. Co-applying MK-801 (10 μM) and NMDA (50 μM) through the bath for 3 min produced a complete and long-lasting NMDA receptor blockade (Supplementary Fig. S1a). Under these conditions, AP5 still reversibly potentiated GABA currents. Importantly, both currents evoked by GABA in the absence and presence of AP5 were blocked by the addition of the GABA_A_R antagonist bicuculline (10 μM) (Supplementary Fig. S1a,  *n* = 3). The AP5-induced potentiation was associated with an increased slope of the current-voltage (I-V) curve without altering the reversal potential (Supplementary Fig. S1b), indicating this potentiation is not voltage dependent (*n* = 3). Taken together, these results strongly suggest that activation of glutamate binding sites on native GABA_A_Rs can positively modulate the receptor function in neurons.

GABA_A_R-mediated inhibitory processes in the CNS include phasic and tonic inhibitions. The phasic inhibition is primarily mediated by the activation of postsynaptic GABA_A_Rs and can be evaluated by recording miniature inhibitory postsynaptic currents (mIPSCs; Fig. 2c). Bath application of AP5 (200 μM) enhanced mIPSC amplitudes (Fig. 2c; control: 29.0 ± 2.9 pA vs AP5: 36.6 ± 3.6 pA, *n* = 7; *p* < 0.01) without changing mIPSC frequency (Fig. 2c; 0.89 ± 0.27 Hz vs 0.90 ± 0.29 Hz, *n* = 7, *p* > 0.05) or other kinetics of mIPSCs (Fig. 2c, Left bottom panel), suggesting that this mIPSC potentiation is primarily a result of modulation of the postsynaptic GABA_A_Rs, rather than a presynaptic alteration of GABA release. The tonic GABAergic inhibition is mainly mediated by the activation of extrasynaptic GABA_A_Rs by low concentrations of ambient GABA in the extracellular compartment and can be revealed by recording of the change in baseline holding currents produced by blocking GABA_A_Rs.^26,27^ As shown in Fig. 2d, bath application of the GABA_A_R antagonist bicuculline (10 μM) produced a tonic current as indicated by the upward shift in the baseline current trace (Fig. 2d; control: 9.67 ± 1.12 pA; *n* = 7) in the absence of AP5, and this tonic current was significantly increased by the prior addition of AP5 (200 μM) in the bath (Fig. 2d; AP5: 17.28 ± 2.80 pA, *n* = 7, *p* < 0.05 compared with control). Since phasic and tonic inhibitions are thought to be mediated by molecularly and pharmacologically distinct GABA_A_Rs and play distinct roles in controlling neuronal excitability, these results suggest that the glutamate allosteric modulation may be a common phenomenon associated with most, if not all, native GABA_A_Rs and may have an important role in regulating neuronal excitability under physiological and/or pathological conditions.

### The glutamate-binding pocket is formed by critical amino acid residues located at the α+/β− interface of the GABA_A_R

**Fig. 3:**
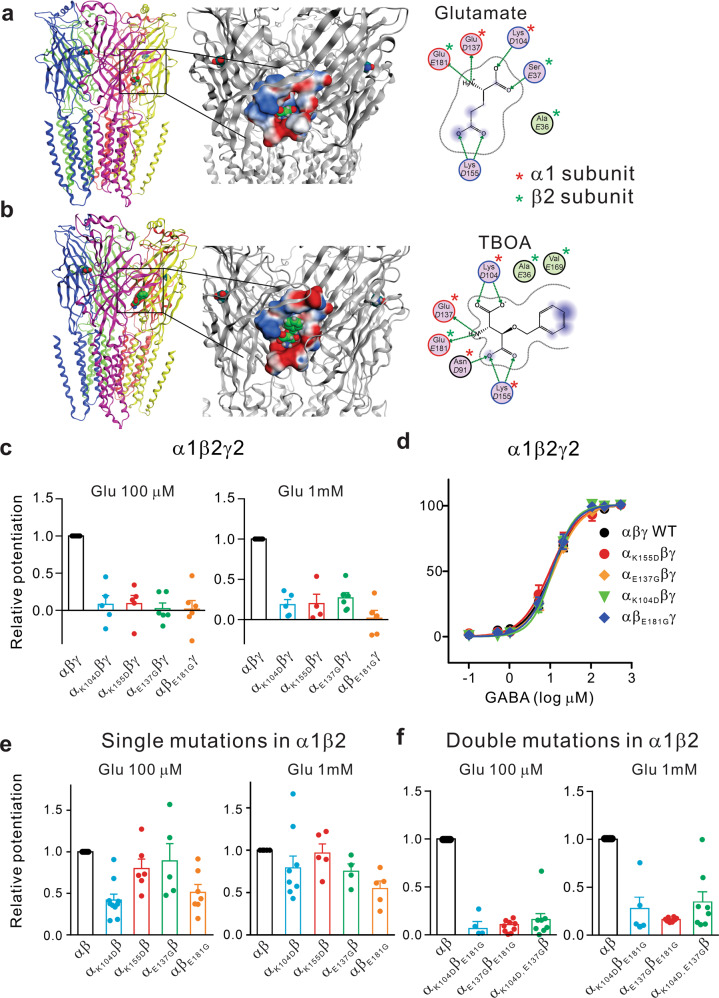
Glutamate binds to the GABA_A_R via a pocket in the α + and β- interface of the receptor. **a**, **b** The putative structures of the glutamate (**a**) or TBOA (**b**) bound α1β2γ2 GABA_A_Rs modeled from the X-ray structures of the glutamate-activated chloride channels are shown on the Left panels. The subunits are color-coded individually. The boxed regions are further enlarged in the middle panels, highlighting the predicted glutamate binding pocket in the α+ and β− interface, just below the loop C. The predicted pocket-forming amino acid residues (particularly, β2_E181_, α1_K104_, α1_K155_ and α1_E137_) and their interactions with glutamate (**a**) or TBOA (**b**) are illustrated in the panels on the right. **c**–**f** Mutational characterization of the putative glutamate-binding pocket in HEK293 cells transiently expressing recombinant human GABA_A_Rs. Relative potentiation was obtained by normalizing potentiation observed in the mutated receptor to that in the respective wild-type receptor. In cells expressing α1β2γ2 GABA_A_Rs, any single mutation of these putative pocket-forming residues was able to impair the glutamate-induced potentiation of GABA (1 µM) currents (**c**; *n* = 5 or 6 in each group), without affecting GABA activation of the receptor (**d**; *n* = 5). However, in cells expressing α1β2 (without a γ2 subunit), the effect of any of the single mutation was much smaller (**e**; *n* = 5 or 6 in each group), and effective elimination of the glutamate potentiation could only be observed with combined mutations of any two of these residues (**e**, **f**; *n* = 5 in each group)

Our results above demonstrate that the glutamate modulation exists in both recombinant and native GABA_A_Rs. Next, we attempted to identify the amino acid residues critical for the glutamate-binding pocket in GABA_A_Rs. Since the glutamate modulation could be observed in the recombinant GABA_A_R containing the α1β2 subunits, and its efficacy was reduced by the introduction of a γ subunit (Fig. 1e, f), we predicted that the glutamate binding site(s) was likely located in α and/or β subunits. We also reasoned that the site may be in an interface region of the two subunits, as the binding site of glutamate on GluCl receptor, which has the similar assembly as GABA_A_Rs and other Cys-loop group receptors, located at such a region.^24^ As glutamate could not compete with GABA (Fig. 1g, h) at the GABA agonist binding sites located at the β+/α− interface,^19^ we further speculated that the glutamate-binding might occur somewhere around the α+/β− interface. With these assumptions in mind, we used an in silicon molecular docking approach to guide our search for the putative glutamate-binding pockets in the GABA_A_R. Using the crystal structures of the glutamate-gated chloride channel as a model,^24^ we generated the computer-based homology modeling of the most common native GABA_A_R which has a subunit composition of two α1, two β2, and one γ2 subunits (Fig. 3a, b). We looked for potential glutamate binding pockets with a particular focus on the extracellular α+/β− interface region. After docking glutamate to this region, we found that there were several potential binding pockets that could potentially accommodate a glutamate molecule at the α+/β− interface (Supplementary Fig. S2a). To further increase the predicting accuracy and thereby decrease the number of possibilities, we also tried to incorporate another glutamate-like ligand TBOA into the modeling (Supplementary Fig. S2a, b). TBOA is at least one hydrophobic benzyl group larger than glutamate (Fig. 3a, b Right panels). Since TBOA mimics glutamate in potentiating GABA_A_R-mediated responses (Fig. 1e), we predicted that the glutamate-binding sites/pockets should also be able to accommodate the larger-sized TBOA and, as such, TBOA docking analysis should help us to exclude the binding pockets that were predicted using glutamate but might be too small to accommodate TBOA. As we expected, this led us to focus our efforts on two potential binding pockets located at the α+/β− interface, one around the loop C (P1) and the other just below the loop C (P2), as respectively indicated in Supplementary Fig. S2b. Similar docking results also can be obtained from the newly resolved α1β3γ2 GABA_A_R structure,^28^ as indicated in Supplementary Fig. S3.

To positively confirm one of these two putative binding sites involved in glutamate binding, we performed a systemic mutational analysis of critical amino acids around these two sites followed by electrophysiological characterizations of their impacts on glutamate potentiation in HEK293 cells expressing wild-type or mutated α1β2γ2 GABA_A_Rs (Table 1). As summarized in Table 1a, we found that mutations of amino acids surrounding the putative loop C pocket (P1; Supplementary Fig. S2b) had either no obvious effect on glutamate-induced potentiation or produced a significant reduction in GABA evoked currents. The data clearly suggests that the loop C pocket is not the binding site by which glutamate produces allosteric potentiation of GABA_A_R function. In great contrast, mutating any of the five amino acids surrounding the putative glutamate binding pocket just below the loop C (P2; Supplementary Fig. S2b, c; Table 1b) largely eliminate the potentiation of GABA currents by glutamate at both low (100 µM) and high (1 mM) concentrations (Table 1, Fig. 3c and Supplementary Fig. S6a; Glu 100 μM: α_K104D_βγ: 8.0 ± 11.7% of control, *n* = 5; *p* < 0.01; α_K155D_βγ: 9.0 ± 10.9% of control, *n* = 5; *p* < 0.01; α_E137G_βγ: 2.1 ± 7.6% of control, *n* = 6; *p* < 0.01; αβ_E181G_γ: 1.6 ± 11.6% of control, *n* = 6; *p* < 0.01; Glu 1 mM: α_K104D_βγ: 18.6 ± 6.3% of control, *n* = 5; *p* < 0.01; α_K155D_βγ: 19.8 ± 11.7% of control, *n* = 4; *p* < 0.01; α_E137G_βγ: 26.9 ± 6.4% of control, *n* = 6; *p* < 0.01; αβ_E181G_γ: 1.8 ± 9.6% of control, *n* = 5; *p* < 0.01). Importantly, none of these mutations affected the ability of GABA to activate GABA_A_R (Table 1b and Fig. 3d). These results demonstrate that these residues are critically required for glutamate-induced allosteric potentiation of GABA_A_Rs. Thus, taken together, the mutational analysis confirms that modeling predicted residues located below the loop C at the α^+^/β^−^ interface (Fig. 3a, b, Supplementary Figs. S2, S3) are responsible for the glutamate allosteric modulation of GABA_A_R function.

**Table 1:** Mutational analyses identifying the glutamate binding pocket at the α1 + /β2- interface of the α1β2γ2 GABA_A_R

a. Effects on glutamate potentiation by individual mutation of putative α1 or β2 amino acid residues forming the loop C pocket (P1; see Supplementary Fig. S2b)					

	Relative potentiation	EC50 (μM)	Hill Slope	*I*_max_ (nA)	*N*
α1 β2 γ2 WT	100.0%	10.4 ± 1.1	1.41 ± 0.14	1.2 ± 0.4	6
α1^S158A^ β2 γ2	106.9 ± 39.8%	6.4 ± 1.1	1.50 ± 0.21	1.3 ± 0.5	3
α1^Y159A^ β2 γ2	90.8 ± 26.7%	7.9 ± 1.1	1.45 ± 0.25	1.1 ± 0.5	3
α1^S205A^ β2 γ2	113.1 ± 33.0%	10.2 ± 1.0	1.16 ± 0.43	2.3 ± 1.1	3
α1^T206A^ β2 γ2	129.3 ± 61.7%	8.6 ± 1.3	1.50 ± 0.45	1.9 ± 0.8	3
α1^E208A^ β2 γ2	90.9 ± 28.7%	9.4 ± 1.3	1.46 ± 0.53	1.8 ± 0.8	3
α1 β2^G126A^ γ2	n/a	152.7 ± 1.1	2.28 ± 0.74	1.3 ± 0.4	3
α1 β2^Q63A^ γ2	n/a	163.4 ± 1.2	2.09 ± 0.93	1.0 ± 0.5	3
α1^H101A^ β2 γ2	n/a	120.0 ± 1.2	1.45 ± 0.52	0.9 ± 0.5	3
α1^F99A^ β2 γ2	96.4 ± 23.0%	8.9 ± 1.3	1.10 ± 0.27	1.0 ± 0.7	3
α1 β2^V177A^ γ2	89.5 ± 28.3%	8.7 ± 1.1	1.12 ± 0.37	1.7 ± 0.9	3
α1 β2^Y61A^ γ2	n/a	176.5 ± 1.2	2.91 ± 1.30	1.0 ± 0.5	3

b. Effects on glutamate potentiation by individual mutation of putative α1 or β2 amino acid residues forming the pocket below loop C (P2; see Supplementary Fig. S2b, c)					

	Relative potentiation	EC50 (μM)	Hill Slope	*I*_max_ (nA)	*N*
α1^K104D^ β2 γ2	8.0 ± 10.7%	11.0 ± 1.1	1.70 ± 0.17	2.3 ± 0.7	6
α1^E137G^ β2 γ2	2.1 ± 7.7%	11.9 ± 1.0	1.35 ± 0.03	1.2 ± 0.3	6
α1^K155D^ β2 γ2	9.0 ± 10.0%	8.5 ± 1.1	1.39 ± 0.21	1.3 ± 0.5	6
α1 β2^E181G^ γ2	1.6 ± 11.6%	10.0 ± 1.1	1.59 ± 0.14	1.1 ± 0.2	6
α1 β2 ^I180A^ γ2	18.8 ± 12.9%	8.0 ± 1.2	1.42 ± 0.27	1.7 ± 0.4	6

HEK cells were transiently co-transfected with either wild or mutant α1, β2, and γ2 subunits. Whole-cell recordings were performed with a Cl-based recording pipette at a holding membrane potential of −60mV, and GABA currents were evoked with fast perfusion of GABA (1 µM; 3 s). Glutamate potentiation were determined by co-applications of glutamate 100 μM; and normalized to that observed in HEK cells expressing wild-type α1β2γ2 receptors

As the putative binding pocket is located at the α^+^/β^−^ and does not involve any amino acid residue from the γ subunit, we then performed further mutational analysis in HEK293 cells expressing α1β2 GABA_A_Rs. We found that a single mutation of any of those glutamate-binding pocket-forming amino acid residues in the receptor of this subunit composition could only produce either partial inhibition or no effect on the glutamate potentiation (Fig. 3e and Supplementary Fig. S6b; Glu 100 μM: α_K104D_β: 48.8 ± 9.5% of control, *n* = 6; *p* < 0.05; α_K155D_β: 76.3 ± 10.5% of control, *n* = 6; *p* > 0.05; α_E137G_β: 109.1 ± 30.2% of control, *n* = 5; *p* > 0.05; αβ_E181G_: 51.2 ± 9.5% of control, *n* = 7; *p* < 0.05; Glu 1 mM: α_K104D_β: 94.7 ± 20.7% of control, *n* = 5; *p* > 0.05; α_K155D_β: 90.1 ± 11.3% of control, *n* = 5; *p* > 0.05; α_E137G_β: 88.4 ± 5.9% of control, *n* = 5; *p* > 0.05; αβ_E181G_: 50.6 ± 8.4% of control, *n* = 6; *p* < 0.05), and that a complete elimination of the potentiation required a combination of simultaneously mutating two of the five amino acids (Fig. 3f). More specifically, single mutation at α1K155 (α_K155D_) or E137 (α_E137G)_) produced little effect (Fig. 3e; Glu 100 μM: α_K155D_β: 79.8 ± 11.4% of control, *n* = 6, *p* > 0.05; α_E137G_β: 109.1 ± 30.2% of control, *n* = 3; Glu 1 mM: α_K155D_β: 96.7 ± 10.8% of control, *n* = 5, *p* > 0.05; α_E137G_β: 88.4 ± 5.9 % of control, *n* = 2, *p* > 0.05), whereas mutation of α1 at K104 (α_K104D_) or β2 at E181 (β_E181G_) resulted in a partial, but significant reduction in the potentiation of GABA responses by low concentration (100 μM) of glutamate (Fig. 3e; Left; α_Κ104D_β: 50.8 ± 9.5% of the wild type receptor, *n* = 6, p < 0.05; αβ_E181G_: 51.2 ± 9.4%, *n* = 7; *p* < 0.05). However, when the glutamate concentration was increased to 1 mM, only β2_E181G_ produced partial, but significant reduction of the potentiation (Fig. 3e, Right; α_K104D_β: 99.5 ± 20.9%, *n* = 5; *p* > 0.05; αβ_E181G_: 50.6 ± 8.4%, *n* = 5; *p* < 0.05). These results imply that β2 E181 may be critical for the receptor’s binding with glutamate, and that the less impaired interaction between glutamate and the receptor by a single mutation at α1 K104, or E137, or K155 is possibly due to the partial compensation by β2 E181.

To further investigate the characteristics of these four residues, double amino acid substitutions were introduced at residue pairs that are located at different (α1β2) subunits (Fig. 3f and Supplementary Fig. S6b). Accordingly, mutations of α1 E137G and β2 E181G dramatically reduced the sensitivity to glutamate at both 100 μM (Fig. 3f; α_E137G_β_E181G_: 9.2 ± 3.0% of control, *n* = 4; *p* < 0.05) and 1 mM (Fig. 3f; α_E137G_β_E181G_: 16.2 ± 1.3% of control, *n* = 3; *p* < 0.05). Co-expression of α1 K104D and β2 E181G also strongly decreased the sensitivity to glutamate at both 100 μM (Fig. 3f; α_K104_β_E181G_: 6.6 ± % of control, *n* = 5; *p* < 0.05) and 1 mM (Fig. 3f; α_K104_β_E181G_: 27.4 ± 12.6% of control, *n* = 5; *p* < 0.05). The double α1 mutation at K104D and E137G decreased receptor sensitivity to 100 μM (Fig. 3f; α_K104D,E137G_β: 17.3 ± 12.9% of control, *n* = 5; *p* < 0.05) and 1 mM glutamate (Fig. 3f; α_K104D,E137G_β: 45.9 ± 20.9% of control, *n* = 5; *p* < 0.05). The requirement of double mutations in α1β2 (Fig. 3e, f), but only single mutation in α1β2γ2 receptors (Fig. 3c) is in a good agreement with the electrophysiological results that glutamate produced a more pronounced potentiation in HEK293 cells expressing α1β2 GABA_A_Rs (Fig. 1e) than that expressing α1β2γ2 receptors (Fig. 1f). It provides further support for the modeling predicting glutamate binding pocket (P2) at the α+/β− interface: it is encompassed by 5 amino acids listed in Table 1, and particularly, the four charged residues (K104, K155, E137 on α1, and E181 (to a lesser degree I180) on β2 subunit), that respectively interact with COO^−^ and NH_3_^+^ groups of both TBOA and glutamate (Fig. 3a, b; Supplementary Figs. S2c, S3c).

**Fig. 4:**
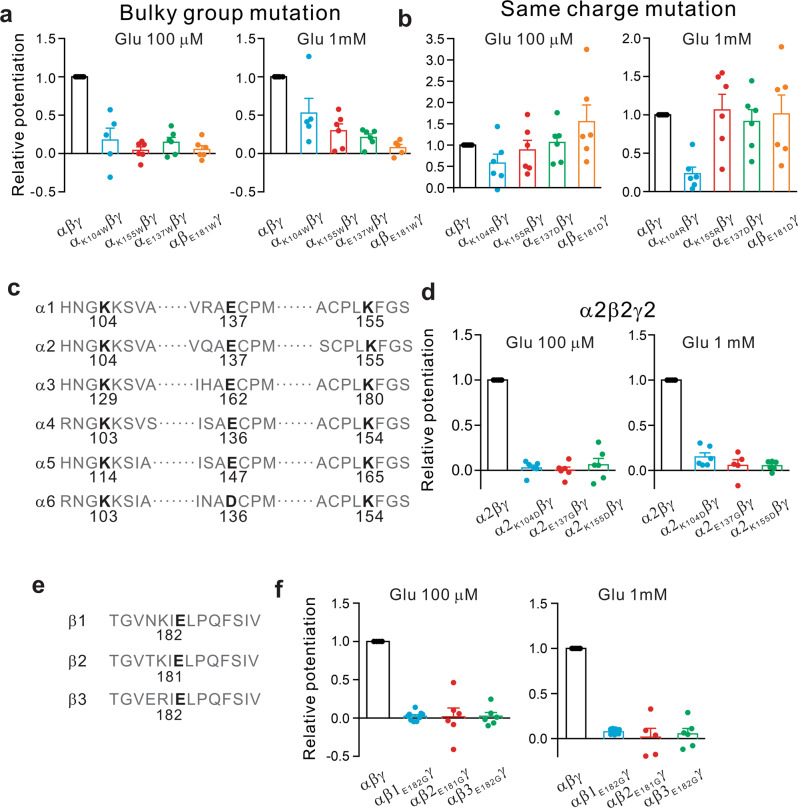
Property characterization of glutamate binding sites in HEK cells expressing wild-type or mutated αβγ GABA_A_Rs. **a**, **b** The effects of electrostatic charge and/or size of the pocket-forming residues on glutamate-induced functional modulation. Substitution of the residue α1_K104_, α1_K155_, α1_E137_, or β2_E181_ with non-charged, bulky tryptophan (W) residue impaired glutamate-mediated potentiation (**a**; *n* = 5 or 6 in each group), whereas mutation of these residues into a different residue with the same charge (except for the K-R mutation) had little effect on the potentiation (**b**; *n* = 5 or 6 in each group). **c**, **d** Residues critical for glutamate binding pocket in α1 are conserved among all α subunits. Sequence alignment showed that α1_K104_, α1_K155_, and α1_E137_ are conserved in all six α subunits (**d**), and mutating either of these conserved residues in α2 similarly impaired the glutamate potentiation (**d**; *n* = 6 in each group). **e**, **f** The critical residue β2E181 is conserved among all β subunits. Sequence alignment showed that β2E181 is conserved (**e**), and mutations of β1_E182G_, β2_E181G_, and β3_E182G_ equally impaired glutamate-mediated potentiation in respective receptors (**f**; *n* = 6 in each group)

To further determine the relative significance between the electrostatic and the side chain steric arrangements of these critical amino acid residues in their interaction with glutamate, we next studied the effects of substitution of these amino acids with amino acids of various sizes or charges on the glutamate potentiation. We substituted α1K104, α1E137, α1K155, or β2E181 with tryptophan, a non-charged amino acid, in order to perturb the electrostatic accessibility of glutamate to the binding pocket. This greatly reduced glutamate potentiation of the GABA currents in HEK293 cells expressing α1β2γ2 GABA_A_Rs (Fig. 4a and Supplementary Fig. S6c; Glu 100 μM: α_K104W_βγ: 17.8 ± 15.3 % of control, *n* = 5; *p* < 0.01; α_K155W_βγ: 4.0 ± 4.7% of control, *n* = 6; *p* < 0.01; α_E137W_βγ: 14.7 ± 6.3% of control, *n* = 6; *p* < 0.01; αβ_E181W_γ: 5.4 ± 4.9% of control, *n* = 6; *p* < 0.01; Glu 1 mM: α_K104W_βγ: 50.8 ± 17.2% of control, *n* = 5; *p* < 0.05; α_K155W_βγ: 29.9 ± 8.8% of control, *n* = 6; *p* < 0.01; α_E137W_βγ: 21.1 ± 4.4% of control, *n* = 6, *P* < 0.01; αβ_E181W_γ: 7.6 ± 4.3% of control, *n* = 6; *p* < 0.01). By contrast, the substitution of either α1E137, α1K155, or β2E181 with a similarly charged residue failed to significantly affect glutamate-induced potentiation (Fig. 4b and Supplementary Fig. S6c; Glu 100 μM: α_K155R_βγ: 88.4 ± 22.5% of control, *n* = 6; *p* > 0.05; α_E137D_βγ: 105.9 ± 18.3% of control, *n* = 6; *p* > 0.05; αβ_E181D_γ: 154.8 ± 39.5% of control, *n* = 6; *p* > 0.05; Glu 1 mM: α_K155R_βγ: 106.4 ± 20.4% of control, *n* = 6; *p* > 0.05; α_E137D_βγ: 91.5 ± 15.4% of control, *n* = 6; *p* > 0.05; αβ_E181D_γ: 101.5 ± 24.5% of control, *n* = 6; *p* > 0.05). The results strongly support a critical requirement for the electrostatic interactions between those residues and glutamate within the pocket. Collectively, these data confirm that α1K104, α1E137, α1K155, and β2E181 residues play critical roles in forming the glutamate-binding pocket at the α^+^/β^−^ interface of GABA_A_Rs, likely through electrostatic interactions between glutamate and the charged residues surrounding the putative pocket.

It is interesting to note that these electrostatic interactions between charged amino acid residues and glutamate are common features among several glutamate binding pockets recently identified on other glutamate-binding proteins/ion channels/receptors co-crystallization studies.^21–23,25^ As summarized in Supplementary Fig. S4, analysis of the common amino acid residues involved in direct or indirect interactions with glutamate within these glutamate-binding pockets supports a notion that glutamate usually binds to a group of commonly conserved amino acids in the glutamate-binding pockets (Supplementary Fig. S4). In this regard, the glutamate binding pocket on the GABA_A_R we identified here also shares the same characteristics, having the positively and negatively charged residues capable of forming the glutamate binding pocket (Fig. 3a, b). These analyses provide additional support for the identified critical residues forming the glutamate-binding site by which glutamate produces allosteric potentiation of GABA_A_Rs.

We next used sequence alignment and mutational analysis to determine if these newly identified critical residues involved in glutamate modulation, is conserved among other α and β containing GABA_A_Rs. Sequence alignment of α1 to α6 showed that the critical residues K104, E137, and K155 in α1 are conserved among all other 5 α subunits (Fig. 4c). We next used α2 as an example to test the functional conservation of glutamate-induced potentiation of GABA currents. As shown in Fig. 4d and Supplementary Fig. S6d, similar to that observed in α1-containing GABA_A_Rs (Fig. 1f), glutamate was also capable of potentiating GABA currents in HEK293 cells expressing α2/β2/γ2 GABA_A_Rs; and as expected, individual mutation of the conserved critical residues α2K104, α2E137, and α2K155 with either non-charged or oppositely charged residues also abolished glutamate-mediated potentiation of GABA currents in these cells (Fig. 4d and Supplementary Fig. S6d; Glu 100 μM: α2_K104D_βγ: 2.7 ± 3.1% of control, *n* = 6; *p* < 0.01; α2_E137G_βγ: 0.3 ± 3.4% of control, *n* = 6; *p* < 0.01; α2_K155D_βγ: 6.3 ± 6.9% of control, *n* = 6; *p* < 0.01; Glu 1 mM: α2_K104D_βγ: 15.1 ± 4.4% of control, *n* = 6; *p* < 0.01; α2_E137G_βγ: 5.7 ± 6.2% of control, *n* = 5; *p* < 0.01; α2_K155D_βγ: 5.3 ± 2.1% of control, *n* = 6; *p* < 0.01). Similarly, sequence alignment also showed that E181 in β2 was conserved among the other two β subunits; corresponding to E182 in the β1 and β3 subunits (Fig. 4e). In agreement with this sequence conservation, we found that glutamate could also potentiate GABA-induced currents in either α1/β1/γ2 or α1/β3/γ2-expressing HEK293 cells (Fig. 4f and Supplementary Fig. S6d), and most importantly, mutation of E182 in either β1 or β3 prevented glutamate induced potentiation of GABA-induced currents (Fig. 4f and Supplementary Fig. S6d; Glu 100 μM: αβ1_E182G_γ: 2.4 ± 2.0% of control, *n* = 9; *p* < 0.01; αβ3_E182G_γ: 2.3 ± 5.0% of control, *n* = 6; *p* < 0.01; Glu 1 mM: αβ1_E182G_γ: 7.6 ± 1.0% of control, *n* = 9; *p* < 0.01; αβ3_E182G_γ: 2.4 ± 7.9% of control, *n* = 6; *p* < 0.01). Given that most native GABA_A_Rs contain α and β subunits, the results strongly suggest that this glutamate-binding pocket, and hence functional modulation, are conserved in most, if not all, native GABA_A_Rs.

### γ subunit compromises glutamate allosteric potentiation of GABA_A_Rs by reducing the number of glutamate binding pockets

**Fig. 5:**
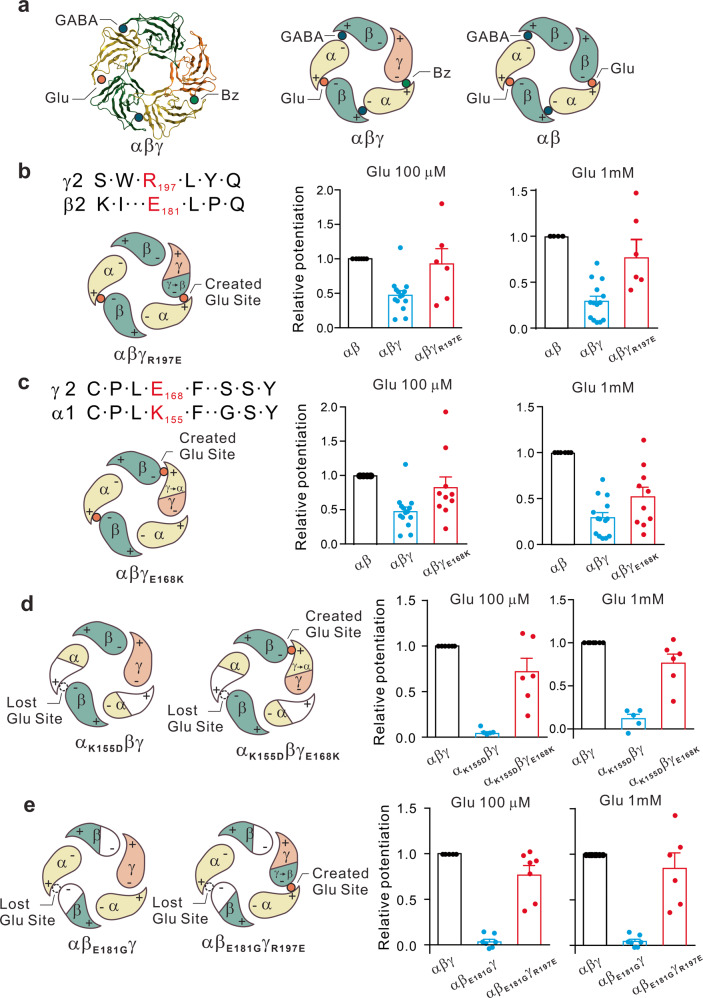
γ subunit reduces the glutamate potentiation efficacy by disrupting a binding pocket in the GABA_A_R. **a** Top-down structural views of pentameric GABA_A_R with α1β2γ2 (Left and Middle panels) and α1β2 (Right panel). GABA (Black dot), glutamate (Glu; Red dot) and benzodiazepine (Bz; Green dot) binding sites are respectively located at different interfaces in αβγ (middle) and αβ GABA_A_Rs (right). The requirement of critical residues for the glutamate-binding pocket in the α+/β− interface predicts that the αβγ receptor contains only one functional glutamate binding site (Left and Middle panels), whereas αβ receptor has two (Right panel). **b**–**e** Mutational analysis in HEK293 cells expressing αβγ GABA_A_Rs confirms the lack of the required residues for the glutamate binding pocket in the γ subunit. **b** The sequence alignment indicates the substitution of β2_E181_ at the corresponding position of γ2 subunit with an opposite charged residue of R197 (Left), preventing the formation of the second glutamate binding pocket. Converting the charge with the γ2_R197E_ mutation creates a new glutamate binding pocket at the α+/γ− interface (α1β2γ2_R197E_; Red dot; left panel), increasing the glutamate potentiation to the level comparable to that of α1β2 receptors (bar graphs on the middle and right panel, *n* = 6 in each group). **c** Similarly, γ2_E168K_ mutation mimics α1_K155_ creating a new glutamate binding pocket at the γ2+/β− interface (α1β2γ2_E168K_; Red dot; Left panel), and thereby increases the glutamate potentiation to the level similar to that of α1β2 receptor (Bar graphs on the middle and right, *n* = 10 in each group). **d** The impaired glutamate potentiation due to the loss of required negative charged residue α1K155 (α_K155D_βγ; Left panel) was fully rescued by the newly created glutamate binding pocket at the γ+/β− interface by γ2_E168K_ (α_K155D_βγ_E168K_; Left panel; and Bar graphs on middle and right panel; *n* = 6 in each group). **e** Similarly, creating a new glutamate binding pocket at the α1 + /γ2- interface with γ2_R197E_ mutation (αβ_E181_γ_R197E_; Left panels) rescues glutamate potentiation deficit due to the loss of the sole glutamate binding site produced with β2_E181G_ (αβ_E181G_γ; Left panels; and Bar graphs on the Middle and Right; *n* = 7 in each group)

Although, above results indicate that the glutamate-binding pocket is formed by critical residues of α and β subunits with no residue from γ subunit, we did find that the presence of a γ subunit in the receptor reduced the efficacy of glutamate-mediated modulation (Fig. 1e, f) and mutational disruption of the binding pocket (Fig. 3c–e). Given the fact that glutamate binding pocket is formed by critical residues of α and β subunits at the α^+^/β^−^ interface, we hypothesized that as shown in Fig. 5a, depending on the presence of a γ subunit or not, the GABA_A_R might have one or two glutamate binding sites, thereby having different sensitivity to glutamate potentiation and mutational disruption. For the GABA_A_Rs containing the αβγ subunits, there is only one α^+^/β^−^ interface and hence can only form a single glutamate-binding pocket (Fig. 5a; Left and Middle). However, for the GABA_A_R containing only α and β subunits, there are two α^+^/β^−^ interfaces, with potentially two glutamate-binding pockets (Fig. 5a; Right). To validate our hypothesis, we first questioned why a γ subunit at α^+^/γ^−^ or γ^+^/β^−^ interfaces cannot form the glutamate binding pocket with their counterparts. Sequence alignment of γ2 and β2 showed that the residue of the γ2 subunit corresponding to the E181 residue of β2 is a positively charged residue R197 (Fig. 5b; Top panel on the left). Given that via its interaction with the positively charged NH^3+^ group of glutamate, the negatively charged β2 E181 is required for the formation of the glutamate binding pocket at the α^+^/β^−^ interface, the oppositely charged γ2 R197 is unlikely able to replace the β2 E181 in forming the glutamate-binding pocket in the α^+^/γ^−^ interface. We therefore further reasoned that if that is the case, then a simple mutational substitution of γ2 R197 with a negatively charged residue Glu (γ2_R197E_) should be able to create a new site at the α^+^ and γ^−^ interface (Fig. 5b; Bottom panel on the left), thereby restoring the level of glutamate potentiation in αβγ receptors to that observed in αβ receptors. Indeed, as we expected, co-expression of γ2_R197E_ with wild type α1 and β2 subunits increased glutamate-induced potentiation of GABA currents to a level comparable to that observed in α1β2 receptors at both glutamate concentrations of 100 μM (Fig. 5b; Middle panel, Glu 100 μM-induced potentiation on α1β2γ2_R197E_: 92.7 ± 22.0% of wild-type α1β2, *n* = 6; *p* > 0.05, 204.9 ± 48.7% of wild-type α1β2γ2, *n* = 6; *p* < 0.05) and 1 mM (Fig. 5b; Right panel Glu 1 mM-induced potentiation on α1β2γ2_R197E_ potentiation: 82.4 ± 16.9% of wild-type α1β2, *n* = 6; *p* > 0.05; 190.9 ± 39.2% of wild-type α1β2γ2, *n* = 6; *p* < 0.05). Similarly, alignment of γ2 and α1 showed that γ2 contains a negatively charged residue (E168) at the position corresponding to the positively charged α1 K155 that is required for forming glutamate binding pocket along with the β subunit in the α^+^/β^−^ interface (Fig. 5c, left). Importantly, mutating this residue into a positively charged K residue in γ2 (γ2_E168K_) was able to create an additional glutamate-binding site at the γ^+^/β^−^ interface and thereby increased glutamate potentiation of α1β2γ2_E168K_ to the level comparable to that of α1β2 receptors (Fig. 5c, Middle and right panels and Supplementary Fig. S6e; Glu 100 μM-induced potentiation on α1β2γ2_E168K_: 169.4 ± 32.5% of wild-type α1β2γ2, *n* = 10; *p* < 0.05; Glu 1mM-induced potentiation α1β2γ2_E168K_: 225.7 ± 51.9% of wild-type α1β2γ2, *n* = 10; *p* < 0.05). The γ2_E168K_ mutation partially restored the compromised potentiation in γ2-containing receptors (Fig. 5c, Middle and right panels and Supplementary Fig. S6e), indicating that the positive charge of the side chain of residue at γ2 168 position is crucial to form the glutamate binding site at the γ^+^/β^−^ interface which can mimic K155 to form the glutamate binding site at the α^+^/β^−^ interface.

As mentioned above, we demonstrated that a single mutation of these pocket-forming amino acid residues of either α1 or β2 in α1β2γ2 receptors (Fig. 3c), but not in α1β2 receptors (Fig. 3e), is sufficient to eliminate glutamate modulation. This is likely due to the fact that the single mutation is sufficient to disrupt the sole glutamate-binding pocket formed at the single α^+^/β^−^ interface of a γ-containing receptor (Fig. 5a). If that is the case, as illustrated in the Left two panels of Fig. 5d, we predicted that by creating a new glutamate binding pocket at γ^+^/β^−^ with the γ2 mutations described above (Fig. 5b, c), we should be able to restore glutamate sensitivity to α or β mutation. We therefore tested if we could rescue the loss in glutamate potentiation by the single α1 K155D in α1β2γ2 (Fig. 5d; left panel; α1_K155D_β2γ2) with the creation of a new glutamate-binding site at γ^+^/β^−^ interface by introducing the γ2 E168K mutation (mimicking glutamate pocket forming residue α1K155) into the receptor (Fig. 5d; left panel; α1_K155D_β2γ2_E168K_). As shown in the right panels of Fig. 5d, introducing the γ2 E168K mutation was able to partially restore the glutamate sensitivity at both 100 μM glutamate (Fig. 5d; middle panels and Supplementary Fig. S6e: restored potentiation to 71.8 ± 14.7% of wild-type α1β2γ2, in the presence of γ2_E168K_ mutation, *n* = 6) and 1 mM glutamate concentrations (Fig. 5d; right panels and Supplementary Fig. S6e; restored potentiation to 76.4 ± 10% of wild-type α1β2γ2, in the presence of γ2_E168K_ mutation, n = 6). Similarly, creating a new glutamate-binding pocket at α^+^/γ^−^ with the γ2 R197E mutation in the glutamate modulation deficient α1β2_E181G_γ2 receptor (Fig. 5e; left panels) also partially rescued the β2 E181G mutation-induced deficit in glutamate potentiation (Fig. 5e, middle and right panels and Supplementary Fig. S6e; middle panel: Glu 100 μM: restored potentiation to 76.6 ± 10.4% of wild-type α1β2γ2, in the presence of γ2_R197E_ mutation, *n* = 7; Right panels: Glu 1 mM: restored potentiation to 84.5 ± 16.9% of wild-type α1β2γ2, in the presence of γ2_R197E_ mutation, *n* = 6). Taking together, these results clearly demonstrate that glutamate exert an allosteric potentiation of GABA_A_R function by direct binding to the glutamate-binding pocket(s) formed with several charged amino acid residues of α and β subunits at the α^+^/β^−^ interface of the GABA_A_R.

### Glutamate allosteric potentiation of GABA_A_Rs functionally important in intact animals under both physiological and pathological conditions

**Fig. 6:**
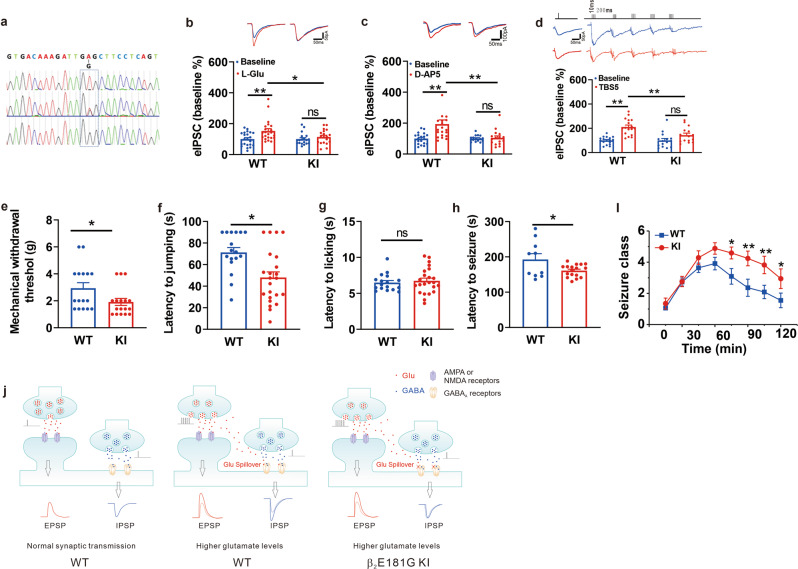
β2_E181G_ knock-in (KI) mice have impaired glutamate potentiation and exhibit phenotypes of increased neuronal excitability. **a** DNA genotyping confirms the correct GAG-GGG mutation that converts glutamic acid at 181 residue of β2 subunit of WT mice into glycine (β2E181G) in both heterozygous (Het) and homozygous (Hom) mice. **b**, **c** Homozygous β2_E181G_ KI mice reveal the impairment of glutamate potentiation of GABA_A_R responses. Whole-cell voltage clamp recording of CA1 neurons showed that glutamate (**b**; L-Glu (Red), 50 μM; WT, *n* = 22; KI, *n* = 19) or glutamate-like ligand AP5 (**c**; AP5 (Red), 50 μM; WT, *n* = 20; KI, *n* = 16) potentiates currents evoked by micropressure-injection of GABA (10 μM) in hippocampal slices of wildtype, but not in slices of β2_E181G_ homozygous KI mice. **d** Potentiation of IPSCs by theta-burst stimulation (TBS; top panel in **d**) is significantly reduced in slices from KI mice (n = 15) in comparison with that in slices from WT mice (*n* = 17). The amplitudes of IPSCs evoked by TBS are normalized to their own IPSC evoked with a single pulse. **e**–**g** Homozygous KI mice have decreased thresholds to both pressure (*n* = 16 for each group; **e**) and temperature (*n* = 17 for WT; *n* = 23 for KI; **f** and **g**) stimuli to the limbs. **h**, **i** Homozygous KI mice exhibit increased seizure susceptibility. Comparing with WT mice (*n* = 10), KI mice (*n* = 17) had significantly shortened latency and increased severity of the kainic acid (20 mg/kg; i.p.)-induced seizure activity. **j** Proposed model for a homeostatic feedback role of glutamate potentiation of GABAAR function. Left Panel: under basal conditions, efficient synaptic uptake mechanisms ensure the presynaptically released glutamate to be mainly restricted within the cleft of glutamatergic synapses without any significant spill-over onto adjacent GABAergic synapses. Thus, glutamate mainly functions as a specific excitatory transmitter to generate normal levels of excitatory postsynaptic potentials at the glutamatergic synapse. Middle Panel: However, under certain physiological or pathological conditions, a significantly increased glutamate release and/or compromised glutamate uptake may cause a significantly increase in the glutamate concentration at the cleft of excitatory synapses that surpasses the synaptic uptake capacity. Under these conditions, the increased concentration of glutamate, while resulting in the increased EPSP amplitude at excitatory synapses, spills over to adjacent GABAergic synapses, thereby simultaneously increasing GABA_A_R-mediated postsynaptic inhibitory potentials (IPSCs). By doing so, this newly discovered glutamate-GABA_A_R feedback mechanism ensures the excitation-inhibition balance and hence neuronal excitability is largely maintained. Right Panel: In KI mice, the loss of such a homeostatic feedback mechanism leads to an unchecked increase in the excitatory synaptic transmission (EPSP amplitude), resulting in excitation-inhibition imbalance, and hence heightened neuronal excitability under conditions of an increased presynaptic glutamate release and/or compromised glutamate uptake

Our mutational analysis identifies the glutamate binding pockets at the α+ and β− interfaces of the GABA_A_R (Fig. 5a). Although the EC50 of glutamate modulation appears to be much higher than the basal extracellular glutamate concentrations, it can be reached under certain physiological and pathological conditions, such as high-frequency stimulation of presynaptic inputs, seizure activity, or brain ischemia. We therefore hypothesize that under these specific conditions, this glutamate modulation could function as a negative feedback mechanism to restrain overexcitation, which has critical physiological and pathological significance. Due to the lack of a specific antagonist for this novel glutamate binding on the GABA_A_R, we next examined its physiological and pathological roles in intact animals by generating knock-in (KI) mice carrying glutamate modulation deficient GABA_A_Rs. The β subunit is an obligatory subunit that is required for the formation of functional GABA_A_Rs. The β2 subunit is one of the most widely expressed subunits in mammalian brain and most importantly, we found that a single E181G mutation in the subunit could abolish glutamate modulation without affecting the GABA activation of the receptor (Fig. 3c, d). Therefore, we reasoned that KI mice carrying β2_E181G_ mutation would have significantly reduced glutamate potentiation in majority of the native GABA_A_Rs, thereby exhibiting overexcitation phenotypes. We generated KI mice carrying the β2_E181G_ mutation. The successful generation of β2_E181G_-KI was confirmed with DNA genotyping (Fig. 6a). As we predicted, both hetero- and homozygous mice of these KI lines are fully fertile, albeit growing at a lightly slow rate (there was an about 20% weight loss in homozygous KI mice in comparison with their WT counterparts).

We next determined if glutamate-mediated allosteric potentiation of GABA_A_R function was impaired in hippocampal slices acutely prepared from postnatal 90-day-old KI mice. Whole-cell recordings of CA1 neurons were performed under voltage clamp configuration at a holding potential of −60 mV. KI mice did not affect the basic properties of inhibitory synaptic transmission (Fig. S7). To overcome the powerful glutamate uptake capacity in slices, we applied glutamate at a high concentration (50 µM) at which it significantly potentiated GABA_A_R-mediated IPSCs currents in slices from the WT mice (Fig. 6b, WT: L-Glu, 153.0 ± 9.5% relative to baseline, *n* = 22; *p* < 0.01). On the contrary, glutamate at the same concentration (50 µM) failed to alter eIPSCs in slices from KI mice (Fig. 6b, KI: L-Glu, 114.2 ± 9.9% relative to baseline, *n* = 19; *p* > 0.05; *p* < 0.05, KI vs. WT). To rule out the potential requirement of glutamate receptor activation in glutamate-induced potentiation, we supplemented the glutamate results with AP5, an N-methyl-D-aspartate receptor antagonist that has been shown to be an effective agonist for the glutamate-binding site on GABA_A_Rs. As shown in Fig. 6c, bath application of AP5 (50 µM), while reliably potentiating GABA-evoked currents in slices from the WT mice (Fig. 6c; WT: 193.5 ± 27.0% relative to baseline, *n* = 20; *p* < 0.001), failed to increase the currents in slices from the homozygous KI mice (Fig. 6c; KI: 104.6 ± 12.7% relative to baseline, *n* = 16; *p* > 0.05; *p* < 0.01, KI vs. WT).

To test the allosteric potentiation of GABA_A_Rs by presynaptically released glutamate under certain physiological conditions, we delivered five trains of four pulses of theta-burst stimulation to increase endogenous glutamate released from the presynaptic glutamatergic terminals, and compared its effect on the pharmacologically isolated eIPSCs (following blockade of both AMPAR and NMDAR-mediated EPSCs) in WT and KI mice. KI mice did not affect presynaptic release (Fig. S8). As shown in Fig. 6d, e, the summated amplitude of IPSCs evoked by TBS, which were normalized to their own baseline IPSCs evoked with a single pulse, were significantly larger in slices from the WT mice than that in slices from KI mice; thus, the TBS is increased in slices from WT mice (Fig. 6d; WT: Black bars, 210.7 ± 16.1%, *n* = 17) in comparison with that in slices from KI mice (Fig. 6d; KI: Red bars, KI: 143.9 ± 16.3%, *n* = 15; *p* < 0.01, KI vs. WT). These results confirmed the significant reduction in the glutamate allosteric potentiation of GABA_A_R function in the β2_E181G_ KI mice. Supporting the predicted negative feedback role of the glutamate-GABA_A_R crosstalk in controlling neuronal excitability, we found that the KI mice, in comparison with their WT counterparts, had a significantly increased neuronal excitability as reflexed by the reduction in the thresholds to noxious, both mechanical (Fig. 6e; WT: 2.9 ± 0.4 g, *n* = 16; KI: 1.9 ± 0.3 g, *n* = 17; *p* < 0.05) and temperature, stimulations (Fig. 6f, g; WT: 71.5 ± 4.5 s, *n* = 17; KI: 48.0 ± 5.2 s, *n* = 23; *p* < 0.01). We also further confirmed the increased neuronal network excitability phenotype of the KI mice using a well-characterized mouse model of epilepsy induced with i.p. injection of kainate acid (20 mg/kg; i.p.). We found that there was a significant reduction in the latency (Fig. 6h; WT: 189.6 ± 15.2 s, *n* = 10; KI: 161.0 ± 4.0 s, *n* = 17; *p* < 0.05) and increase in the severity of kainic acid-induced seizure activity in KI mice in comparison with their WT counterparts (Fig. 6i; *p* < 0.01). All these results highlight the physiological and pathological significance of this novel glutamate-GABA_A_R crosstalk as a homeostasis-negative feedback mechanism in fine-tuning synaptic excitation-inhibition balance, thereby ensuring a normal level of neuronal excitability.

## Discussion

In the present study, we identified a novel glutamate binding site in the GABA_A_Rs, thereby revealed a previously un-appreciated crosstalk between the excitatory transmitter glutamate and inhibitory GABA_A_Rs. Through a detailed ligand-binding and molecular characterization in recombinant expression system, we demonstrated that this cross-talk is mediated by an allosteric potentiation of GABA_A_Rs through a direct binding of glutamate to the GABA_A_R itself. Genetic elimination of this cross-talk in mice allowed us to conclude that this cross-talk functions as a negative feedback, thereby having significant physiological and/or pathological roles in fine-tuning synaptic excitation-inhibition balance. Along with the recently reported glutamate modulation of glycine receptor,^10^ the present study strongly suggests that such a positive modulation of inhibitory receptor-gated chloride channels by excitatory transmitter glutamate may be a common feedback mechanism between synaptic excitation and inhibition, being of physiological and pathological significance.

With [3H]glutamate binding assays, we were not only able to confirm the direct binding of glutamate to GABA_A_Rs, but also able to demonstrate that the glutamate binding site does not overlap with known GABA agonist binding sites. A combination of computer-assisted in silicon docking and mutational analysis allowed us to positively identify a novel glutamate binding pocket located in the α+/β− interface, involving critical amino acid residues of K104, E137, and K155 of α1 subunit and E181 of β2 subunit (Fig. 3a, b). The critical amino acid residues involved in the formation of the pockets are very similar to glutamate-binding pockets recently identified in other glutamate binding proteins/receptors (Supplementary Figs. S2, S3).^21–25^ These critical amino acid residues include the negatively charged residues (α1E137 and β2E181) for interacting with the positively charged –NH_3_^+^ of glutamate; and the positively charged residues (α1K104 and α1K155) for interacting with the negatively charged –COO^−^ group of glutamate (Fig. 3a, b). With the best-assembled cryo-EM GABA_A_Rs (α1β3γ2) model,^28^ the glutamate and TBOA can also dock in the similar region (Supplementary Fig. S4), and E182 of β3 subunit, which corresponds to the E181 of β2 subunit, is found to be critical for glutamate-mediated potentiation. It is important to note that those critical residues in α and β subunits are conserved among their respective subfamilies (Fig. 4c, e). Since most native GABA_A_Rs contain α and β subunits,^2,29^ the glutamate binding pocket likely exists and functionally operates among most, if not all, native GABA_A_Rs including those localized at the synaptic and extrasynaptic sites, thereby having more profound and wide spread physiological, pathological and therapeutic significances. Consistent with this notion, we were able to demonstrate that glutamate binding site agonist AP5 can potentiate both phasic (synaptic) currents mediated by postsynaptic GABA_A_Rs (Fig. 2c) and tonic currents-mediated by extrasynaptic GABA_A_Rs (Fig. 2d).

Another notable feature of the GABA_A_R glutamate binding pocket is that it is distinct from any other known ligand and modulatory sites on the GABA_A_R. First, it does not overlap with the GABA-binding sites previously identified at the β+/α− interfaces (Fig. 5a),^2^ and this is fully supported by our results that even at much higher concentration, GABA failed to competitively replace glutamate binding in the [3H]glutamate binding assays (Fig. 1h); and that mutational elimination of the glutamate modulation did not affect GABA activation of GABA_A_Rs (Fig. 3d and Table 1). Secondly, this newly identified glutamate-binding pocket is structurally and functionally distinct from the most well-characterized allosteric benzodiazepine modulatory site previously identified at the α+/γ− interface, which requires the presence of a γ subunit in the GABA_A_R.^2,29–31^ By contrast, the glutamate binding site identified in the present study is located the α+/β− interface, and does not require the presence of a γ subunit (Fig. 5a). In fact, incorporation of a γ subunit actually partially impairs the glutamate-induced potentiation (Fig. 1e, f), and this is primarily due to the inability of a γ subunit to form the glutamate binding pocket with either α or β subunit (Fig. 5b–e). It is interesting to note that some benzodiazepine-related chemicals such as CGS9895 can also positively modulate GABA_A_R function via interacting with a putative binding pocket also located in the α+/β− interface^20,32^ (Supplementary Fig. S2c). However, since mutation of the α1-H101 that is critically required for CGS9895^20,32^ failed to affect glutamate potentiation of GABA_A_R function (Table 1), the CGS9895 binding pocket in the α+/β− interface is clearly distinct from the new glutamate-binding pocket identified in the present study.

Our results together revealed an allosteric potentiation of inhibitory GABA_A_R function by the major excitatory transmitter glutamate through a mechanism of glutamate binding to the GABA_A_R. It is important to note that the EC50 of glutamate modulation (Fig. 2b) we observed (~180 µM) is much higher than the basal levels of extracellular glutamate, which are from nM to low µM.^33,34^ In what physiological or pathophysiological circumstance will glutamate-mediated potentiation of GABA_A_R take effect in the CNS? Several studies have demonstrated that glutamate can be co-released with GABA at GABAergic terminals in different brain regions.^11–15^ The co-released glutamate can activate adjacent AMPA receptors, indicating that the co-released glutamate can reach high µM to mM concentrations locally at some inhibitory synaptic clefts. The co-releasing mechanism is proposed to be involved in the pathogenesis of schizophrenia and in the processes of cocaine withdrawal and relapse.^12^ Moreover, under certain physiological and pathological conditions, extracellular glutamate concentrations can increase to the level close to or even above the EC50, thereby engaging the glutamate allosteric potentiation of the adjacent GABA_A_Rs. Such conditions include the increased synaptic activities during the production of certain forms of synaptic plasticity or following ischemic brain insults in which the glutamate uptake by glia is blocked and massive glutamate spills over to adjacent GABA_A_Rs.^34–36^ In addition, extracellular glutamate concentrations can increase the following activation of the two-pore-domain potassium channels TREK-1 in astrocytes.^37^ Glia activated during neuronal synchronization or cortical spreading depression might also release glutamate to the extrasyanptic region.^38,39^ We view the higher EC50 as a specific feature that is critically important for ensuring glutamate to function as an excitatory transmitter mediating synaptic transmissions at vast majority of excitatory synapses under most of the physiological conditions. However, under the conditions of overexcitation of glutamatergic neurons and/or compromised glutamate uptake, high level of extracellular glutamate may potentiate the function of GABA_A_Rs. This cross-talk can be engaged to counteract glutamate receptor-mediated overexcitation through the feedback increase in GABA_A_R-mediated neuronal inhibition (Fig. 6e Middle). Thus, the relatively high EC50 for the glutamate-mediated allosteric potentiation of GABA_A_R may potentially bear physiological and pathological importance. When this glutamate-GABA_A_R crosstalk is compromised, such as by the β2_E181G_ mutation in KI mice, the enhanced synaptic excitation following the increased glutamate concentration cannot be efficiently counteracted by the increased GABA_A_R inhibition, which leads to the phenotypes of hyper-neuronal excitability.

However, directly testing its significance under both physiological and pathological conditions requires a novel antagonist that can specifically prevent glutamate binding to the sites on GABA_A_Rs and thereby prevent glutamate allosteric potentiation of GABA_A_Rs without affecting glutamate activation of glutamate receptors. In the absence of such an antagonist, we experimentally investigated the physiological and pathological engagement of this newly identified glutamate-GABA_A_R feedback crosstalk by generating knock-in mice in which a major population of the native GABA_A_Rs are deficient in this glutamate-GABA_A_R crosstalk. Mice of β2 _E181G_ GABA_A_R subunit knock-in significantly reduced glutamate potentiation of GABA_A_R function without affecting baseline GABA_A_R-mediated synaptic currents. Using this line of mice, we are able to demonstrate that this newly identified glutamate-GABA_A_R crosstalk is functionally significant, and engaged under both physiological conditions as evidence by abnormal phenotypes of sensory process, learning and memory and social interactions; and pathological conditions as evidenced by the increase in kainic acid-induced seizure activity (Fig. 6).

In addition to its physiological and pathological relevance, identification of this novel glutamate-GABA_A_R cross-talk may also have important pharmacological significance. GABA_A_Rs contain targeting sites for various therapeutic drugs including benzodiazepines, barbiturates, and anesthetics.^2,3^ In particular, benzodiazepines have been one of the safest and most popular therapeutics for anxiety, sedation, and as anticonvulsants for the treatment of seizures.^40^ However, their utility has been limited by strict subunit-specificity, dependence, and rapidly declining efficaciousness (i.e., tolerance).^40^ The classic benzodiazepines allosterically modulate GABA-induced synaptic inhibition by binding to the benzodiazepine site located in the interface of α+/*γ−* and therefore require the presence of a γ subunit in the receptor (Fig. 5a).^29,30^ Because most extrasynapatic GABA_A_Rs, such as αβδ-composed receptors, do not contain a γ subunit, they are insensitive to benzodiazepines.^41^ As tonic inhibitions, known to be critical for maintaining neuronal excitability,^26,41^ are primarily mediated by these extrasynaptic, non-γ containing receptors, benzodiazepines have little effect on the tonic inhibition. In great contrast, the novel glutamate binding site we identified in the present study only requires α and β subunits, and is thus present on a vast majority of native GABA_A_Rs in the mammalian brain, including those located extracellularly. As such, this new site may represent a more preferable therapeutic target upon which new GABA_A_R positive modulators with a broader receptor spectrum can be developed. The further co-crystallization of glutamate and GABA_A_Rs will undoubtedly facilitate the development of a novel class of GABA_A_R positive modulators acting at the glutamate-binding site as potentially more effective therapeutics for anxiety, sedation, and epilepsy.

## Materials and methods

### Chemicals

N-methyl-D-aspartate (NMDA), D-2-amino-5-phosphonovaleric acid (AP5), α-amino-3-hydroxyl-5-methyl-4-isoxazole-propionate (AMPA), kainic acid (KA), 6-cyano-7-nitroquinoxaline-2,3-dione (CNQX), threo-β-Benzyloxyaspartic acid (TBOA), and MK-801 were purchased from Torcis (Ellisville, Missouri, US). Glutamate and gamma-Aminobutyric acid (GABA) were purchased from Sigma-Aldrich. Bicuculline methobromide was purchased from Alexis Biochemicals.

### Neuronal culture

Cultured hippocampal neurons were prepared from the brains of D18 fetal Wister rats. Tissues were digested with a 0.25% trypsin solution (Invitrogen) for 25 min at 37 °C, and then mechanically dissociated using a fire-polished Pasteur pipette. Next, the cell suspension was centrifuged at 2500 × *g* for 50 s and the cell pellets were resuspended in DMEM containing 10% Fetal Bovine Serum (FBS; Sigma-Aldrich). Cells were seeded on poly-D-lysine-coated 24-well coverslips at a density of 2.5 × 10^5^ cells/well. Cultures were maintained in a humidified incubator with 5% CO_2_ at 37 °C. After 24 h, plating medium was changed to Neurobasal medium supplemented with B-27 supplement and L-glutamine, and the media changed twice weekly thereafter. Cultured neurons were used for electrophysiological recordings between 10–14 days after plating.

### HEK293 cell culture and transfection

HEK293 cells were cultured in DMEM (Invitrogen) supplemented with 10% fetal bovine serum. Cells were grown to 40–60% confluence and transiently transfected using Lipofectamine 2000 (Invitrogen) with 1:0.5–1 plasmid/lipid ratio. Cells were transfected with a pBK-CMV expression vector containing a rat recombinant GABA_A_R α, β or γ subunits. Co-transections were done with a plasmid ratio of 1:1 αβ and 2:2:1 for αβγ subunit combinations, respectively. pcDNA3.1-GFP was also co-transfected along with GABA_A_R subunits as a transfected marker in order to facilitate the visualization of the transfected cells during electrophysiological experiments. Cells were re-plated on glass coverslips after 15–20 h transfection and were cultured for an additional 15–24 h before whole-cell patch-clamp recordings.

### Site-directed mutagenesis

The site-directed mutagenesis of α, β or γ subunits were performed by using the QuikChange method (Stratagene). All mutant clones were confirmed by DNA sequencing. Wild-type or mutant subunits were transfected in HEK293 cells and subjected to electrophysiology examinations.

### Homology modeling of the GABA_A_Rs and ligand docking studies

#### GABA_A_R modeling

The homology model of the most abundant subtype of the α1β2γ2 GABA_A_R was constructed by using the methods described by Bergmann et al.^42^ This protocol uses the x-ray structure of GluCl co-crystallized with glutamate (PDB code 3RIF) as the primary template for homology modeling. The model was constructed using MODELLER 9v7.^43^ Second homology model of the α1β2γ2 subtype was also built using the recent crystal structure of a human gamma-aminobutyric acid receptor, the GABA_A_R – β3 homopentamer (PDB code 4COF) as the template.^44^ The homology model of α1β3γ2 receptor was constructed by replacing the β2 subunit from the initial α1β2γ2 model with the β3 subunit structure, which was obtained from the recent crystal structure (PDB code 4COF).^44^ This was accomplished by implementing a sequence and structural alignment between the β2 and β3 subunits using Molecular Operating Environment (MOE). Once the two subunits were aligned, the β2 subunit was removed, leaving the β3 in its place to generate the final model of α1β3γ2. The homology model of α1β1γ2 was obtained by using the β3 homopentamer^44^ as the template to construct the model for β1. The β1 homology model was then sequenced and structurally aligned by MOE with the β2 subunit of the α1β2γ2 model initially constructed. By removing the β2 subunit, we were able to generate the final model of α1β1γ2. Similar procedures were also employed to construct the model for the α2β2γ2 receptor. In this case, our α1 homology model was used as the template to generate the α2 structure. Structure validation was performed using VERIFY-3D^45^ on the SWISS-PDB server.

#### Preparation of the protein structures for docking

The above GABA_A_R homology model structures were used for molecular docking studies. For protein structure preparation, all solvent molecules have been deleted and the bond order for the ligands (glutamate and TBOA) and protein have been adjusted. The missing hydrogen atoms have been added, and side chains have been energy-minimized using the OPLS-2005 forcefield, as implemented by Maestro. The ligand binding region has been defined by a box centered at (*x* = 5.2596, *y* = 82.1662, *z* = 75.7904) around the binding site residues. No van der Waals scaling factors were applied and default settings were used for all other adjustable parameters.

#### Ligand preparations and molecular docking

All the compounds were built using MOE. Hydrogen atoms were added after the structures were “washed” (a procedure including salt disconnection, removal of minor components, deprontonation of strong acids, and protonation of strong bases). The following energy minimization was performed with the MMFF94x forcefield, as implemented by the MOE, and the optimized structures were exported into the Maestro suite in SD file format. Docking experiments with glutamate and TBOA (except the model 6HUO with GEMDOCK) were performed using Glide, which includes the Schrodinger Package, Maestro, and interface version 9.0.29.^46^ For docking, standard-precision (SP) docking method was adopted to generate the minimized pose, and the glide scoring function (Glide Score) was used to select the final poses for each ligand.

#### Binding site prediction

The binding site of glutamate on GABA_A_R was predicted via a blind docking protocol using TBOA as a molecular probe. Initially, TBOA molecule was docked in both the α+/β− and β+/α− interfaces of the extracellular portions of GABA_A_R. The binding region for the molecular docking was restricted to the extracellular region of the receptor. No restriction was applied to the docking protocol as it allows a more accurate evaluation of the potential binding sites located on the α+/β− and β+/α− interfaces. The majority of the docking poses were located at the interface of each of these domains. The blind docking protocol produced different binding poses of TBOA in various points along the two interfaces of the GABA_A_R. The TBOA molecule was docked 100 times and each pose was scored based on its binding affinity with surrounding residues. There were a total of five distinct binding sites defined by the blind docking of TBOA which was then further characterized by defining the residues present surrounding the ligand. Each potential binding pocket was evaluated based on two criteria: (1) glide score for each of poses generated for a given binding site; and (2) the pocket had at least 1 positive charged residue of either lysine or arginine and 1 negative charged residue of glutamate or aspartic acid. Glutamate was docked in a similar fashion into these five regions and the top scoring pose was considered to be the most probable binding conformation for glutamate for each of the binding pockets.

### Animals

All experimental procedures with animals were conducted following the guidelines of the Canadian Council for Animal Care and approved by the University of British Columbia Animal Care Committee or the guidelines of Chongqing Science and Technology Commission and approved by the Animal Ethics Committee of Children’s Hospital of Chongqing Medical University. All efforts were made to minimize animal discomfort and to reduce the number of animals used.

### Electrophysiology studies

Whole-cell patch-clamp recordings were performed under voltage-clamp mode using an Axopatch 200B or 1D patch-clamp amplifier (Molecular Devices) on HEK293 cells and cultured neurons. Whole-cell currents were recorded at a holding potential of −60 mV, and signals were filtered at 2 kHz, digitized at 10 kHz (Digidata 1322 A). Recording pipettes (3–5 MΩ) were filled with the intracellular solution that contained (mM): CsCl 140, HEPES 10, Mg-ATP 4, QX-314 5, pH 7.20; osmolarity, 290–295 mOsm. BAPTA (10 mM) was added in the intracellular solution. The coverslips were continuously superfused with the extracellular solution containing (mM): NaCl 140, KCl 5.4, HEPES 10, MgCl_2_ 1.0, CaCl_2_ 1.3, glucose 20, pH 7.4; osmolarity, 305–315 mOsm. GABA-induced currents were either applied by GABA using a two-square barrel glass tubing with a perfusion fast-step system (Warner Instruments). Different concentrations of GABA (0.1 µM to 1 mM) or Glu with GABA (1 µM) were applied to cells transfected with WT or mutated GABA_A_R (*N* = 6–8). Dose–response curves were created by fitting data to Hill equation: *I* = Imax/[1 + (EC_50_/[A])^n^], where *I* is the current, Imax is the maximum current, [A] is a given concentration of agonist, n is the Hill coefficient. For whole-cell recordings of mIPSCs and GABA-evoked currents in cultured neurons, CNQX (10 µM) and TTX (0.5 µM) were added in the extracellular solution to minimize the activation of ionotropic glutamate receptors and voltage-gated sodium channels, respectively. All experiments were performed at room temperature.

Mice were deeply anesthetized with urethane (1.5 g/kg, i.p.) and transcardially perfused with ice cold sucrose hypertonic cutting solution (in mM: KCl 3.0, NaH_2_PO_4_.H_2_O 1.25, NaHCO_3_ 26, Na-vitamin C 0.4, Na-lactate 2.0, Na-pyruvate 2.0, D-glucose 10.0, Sucrose 220, CaCl_2_ 0.1, MgCl_2_ 2, MgSO_4_ 4.0, pH 7.4, 290–300 mOsm/l) prior to decapitation as described previously.^47^ After decapitation, the brain was rapidly removed and transferred to the ice cold cutting solution bubbled with 95% O_2_ and 5% CO_2_, and acute coronal brain slices (400 μm) were prepared on a Leica vibratome (VT1200S, Germany). All brain slices were placed into a standard artificial cerebral spinal fluid (ACSF) (in mM: NaCl 124, KCl 2.8, NaH_2_PO_4_.H_2_O 1.25, NaHCO_3_ 26, Na-vitamin C 0.4, Na-lactate 2.0, Na-pyruvate 2.0, D-glucose 10.0, CaCl_2_ 2, MgSO_4_ 1.2, pH 7.4, 290–300 mOsm/l) incubated at 35 °C for 2 h prior to recording. Field excitatory postsynaptic potential (fEPSP) recordings were performed under current-clamp mode using HEKA EPC10 patch-clamp amplifier (HEKA Electronic, Lambrecht/Pfalz, Germany). The recording solution and electrode internal solution were standard artificial cerebrospinal fluid (ACSF) solution. The fEPSPs were evoked by square-wave stimulations (pulse width, 0.1 ms) at a frequency of 0.033 Hz delivered through ISO-Flex stimulus isolator in constant current model (0.05–0.3 mA). The input-output curve at the hippocampal Schaffer collaterals (SC) from CA3 to CA1 was determined by stimulating from 0 to 350 μA with 50 μA step increment. A paired-pulse facilitation (PPF) experiment was conducted at 30, 50, 70 and 110 ms inter-pulse intervals at a stimulus intensity adjusted to 50% of the maximal response size.

Whole-cell patch-clamp recordings were performed under voltage-clamp mode using an HEKA EPC10 patch-clamp amplifier (HEKA Electronic, Lambrecht/Pfalz, Germany) in brain slices. Whole-cell currents were recorded at a holding potential of −60 mV in CA1 of hippocampus. Recording pipettes (3–5 MΩ) were filled with the intracellular solution that contained (mM): CsCl 140, HEPES 10, K-ATP 4, EGTA 0.5, CaCl_2_ 0.15, MgCl_2_ 4.25, pH 7.20; osmolarity, 290–300 mOsm. The extracellular solution containing (mM): NaCl 140, KCl 5.4, HEPES 10, MgCl_2_ 1.0, CaCl_2_ 1.3, glucose 20, pH 7.4; osmolarity, 305–315 mOsm. Evoked inhibitory postsynaptic currents (eIPSCs) were recorded in the pyramidal neuron of hippocampal CA1 area in response to electrical stimulation of the Schaffer pathway. Recording of eIPSCs was made by bath application of CNQX (20 µM) to minimize the activation of ionotropic glutamate receptors, and was adjusted at a stimulus intensity to evoke about 50% of the maximal IPSCs. After obtained a stable baseline, AP5 (50 µM) or L-glutamate (L-Glu, 50 µM) was added in the extracellular solution to detect the potentiation of GABA_A_R-mediated currents. For whole-cell recordings of miniature IPSCs (mIPSCs) in hippocampal CA1, CNQX (20 µM), AP5 (50 µM) and TTX (0.5 µM) were added in the extracellular solution to minimize the activation of ionotropic glutamate receptors and voltage-gated sodium channels, respectively. All experiments were performed at room temperature.

### [3H]-Glutamate binding assay

For membrane preparation, transfected or non-transfected HEK293 cells were washed twice with cold PBS and harvested by scraping into 5 ml cold PBS. Cells were then centrifuged at 1200 × *g* for 12 min at 4 °C and medium was removed. The washing procedure was repeated twice. Then the cell pellet was re-suspended into 1 ml of 50 mM Tris-HCl buffer (pH 7.4, with protease inhibitor) and homogenized using syringes with 18-G, 21-G and 23-G needles.

To separate the membrane, the homogenate were centrifuged for 20 min at 20,000 × *g* at 4 °C. Then the pellet was re-suspended into 1 ml of 50 mM Tris-HCl buffer, pH 7.4 and centrifuged again. After repeating this procedure for one more time, the pellet was re-suspended into 1 ml of 50 mM Tris-HCl buffer, pH 7.4 and the protein concentration was measured.

To conduct the binding assay, 100 µg membrane enriched preparation was incubated with 40 nM [3H]-Glutamate in a total volume of 0.5 ml in 50 mM Tris-HCl buffer, pH 7.4 for 1 h on ice. For competition assay, 0.4 mM non-labeled glutamate, AP5 or GABA was added with 40 nM [3H]-Glutamate in a total volume of 0.5 ml. Then the reaction was terminated by quickly filtering the solution on Whatman filter paper and washing by 3.5 ml Tris-HCl buffer. Radioactivity was measured in a Beckman liquid scintillation counter.

### Generation of β2_E181G_ knock-in mice

#### In vitro transcription of Cas9 mRNA and guide RNA

Double-nicking strategy was used to minimize off-target effects. Two guide RNA sequences were selected using the Zhang laboratory algorithm (http://crispr.mit.edu/). Cas9 mRNA and guide RNAs were transcribed and purified according to the manufacturer’s instructions. In brief, Cas9 mRNA was prepared by in vitro transcription from the pX330 plasmid (Addgene) linearized with XbaI using the mMessage mMachine T7 Ultra kit (Ambion®) and the MEGAclear kit (Ambion®). To generate the template for sgRNA in vitro transcription, sgRNAs were amplified by PCR from the pX330 plasmid with the forward primer 5′-GCTAATACGACTCACTATAGGGAGGCTGTCACTGGC GTGGAAGTTTTAGAGCTAGAAATAGC and the reverse primer 5′-AAAAAAGCACCGAC TCGGTGCCAC using the MEGAshortscript T7 kit (Ambion®) followed by the MEGAclear kit (Ambion®) for RNA purification.

#### Generation of point-mutation mice

100μl of an RNA solution containing Cas9 mRNA, GuideRNAs (gRNA 1#: GGCGATGACAATGCAGTCAC AGG; gRNA 2#: GATGAGTTTATAATCTACGA TGG) and the synthesized donor ssDNA oligonucleotide (ssODN), including β2_E181G_ mutation (sequence: 5′-ACACAACTGATGACATTGAGTTTTACTGGCGCGGCGAtGAcAAcGCcGTgACAGGAGTGACAAAGATTGgGCTTCCTCAGTTCTCTATCGTAGATTATAAACTCATCACCAAGAAAGTTG-3′) were microinjected into the cytoplasm of fertilized eggs (C57BL/6J) before the eggs were implanted into surrogate C57BL/6J females. After injection, zygotes were cultured in M16 medium, and just as the embryos reached the two-cell stage, they were collected and transferred into the oviductal papillae of C57BL/6J host female mice. After birth, genome DNA was extracted from the tail tips of the pups and subjected to PCR to confirm the mutation. Forward primer (β2-F) 5′-GTTTGCCCTTCTGCCTTCAC-3′ and reverse primer (β2-R) 5′-GGACGCCATGCTTCACCTC-3′ were used for genotyping (producing a 1.8 kb band) of F0 generation. PCR products were purified by agarose gel electrophoresis, and the extracted fragments were sequenced using Sanger gene sequencing. The established F0 mice with correct genotypes were used for F1 breeding. Primers β2-F and β2-R were also used for F1 genotyping. The F1 generation mice were bred again to obtain offspring (heterozygous and homozygous) β2_E181G_ mice, and the F1 mice carrying the heterozygous β2_E181G_ gene were backcrossed to WT C57BL/6 J mice for three generations to remove any incidental mutations. F4 to F8 homozygous β2_E181G_ mice were used for electrophysiological and behavioral assessments. E181G mutation was confirmed by sequence analysis of PCR products in each mouse before the experiment.

### Behavioral studies

Wild-type (WT) and KI mice were housed in plastic cages in a temperature-controlled (21 °C) colony room on a 12 h light/12 h dark cycle, and all electrophysiological and behavioral experiments were conducted during light cycle. Food and water were available ad libitum. The genotype of the mice was confirmed by PCR using DNA from tail tissues. All procedures were performed in accordance with Chongqing Science and Technology Commission guidelines for animal research and approved by the Chongqing Medical University Animal Care Committee.

#### Pain threshold tests

The mechanical withdrawal threshold was determined to evaluate mechanical hyperalgesia using calibrated von Frey filaments (Aesthesio, Danmic, CA, USA). Each animal was placed individually into a transparent plastic cage (8 cm × 9 cm × 8 cm) with a wire-mesh floor to allow insertion of the filament from below. The filament was placed against the plantar surface of the hind paw and acclimatized for not less than 10 min before testing. The measurement was repeated three times at 30 s intervals. The average was taken as the mechanical withdrawal threshold.

Thermal hyperalgesia was assessed by using the Cold/Hot Plate Analgesia Meter (Stoelting, Wood Dale, IL, USA). Mice were placed on the hot plate set to a temperature of 55 ± 0.5 °C. The latency of paw withdrawal from the heat stimulus was measured from the starting to the end point of jumping or licking the hind paw. In order to avoid injury and damage, cut off time for this test was 90 s. Both pain-related behavioral tests were assessed using double-blind procedures.

#### KA-induced seizure

Kainic acid (KA) was dissolved in sterile saline at the concentration of 20 mg/ml. KA (20 mg/kg, i.p.) or same volume of saline as vehicle control was administered by intraperitoneal injection to induce seizures. Diazepam (20 mg/kg, s.c., obtained from Children’s Hospital of Chongqing Medical University) was injected 30 min before the injection of KA. Seizure activity was scored every 15 min for 2 h by a trained observer blind to the genotype or treatment of the mice according to the following scale: 0-no response; 1-immobility and staring; 2-forelimb and/or tail extension, rigid posture; 3-repetitive movements, head bobbing; 4-rearing and falling; 5-continuous rearing and falling: 6-severe clonic-tonic seizures; 7-death. All experiments were performed in accordance with approved institutional animal care guidelines.

### Data analysis

Values are expressed as mean ± SEM (*n* = number of experiments). The two-tailed Student’s test was used for statistical analysis and *P* values < 0.05 were considered statistically significant. The mIPSCs were analyzed the rise time, decay constant, amplitude and frequency with Mini-Analysis (Synaptosoft, Decatur, GA). Other electrophysiological data were analyzed with Clampfit (Molecular Devices).

## Supplementary information

Supplementary Figures

## Data Availability

The data and materials used in the current study are available from the corresponding authors upon reasonable request.
