# Supplementary material for: Glutamate and GABAA receptor crosstalk mediates homeostatic regulation of neuronal excitation in the mammalian brain
Source: Signal Transduct Target Ther. 2022 Oct 3;7:340. doi: 10.1038/s41392-022-01148-y (PMC9527238; doi:10.1038/s41392-022-01148-y)
Supplement: Supplementary file 1 — Supplementary Figures [file 41392_2022_1148_MOESM1_ESM.docx]

**Glutamate and GABA_A_R crosstalk mediates homeostatic regulation of neuronal excitation in the mammalian brain**

Ya Wen^1*^, Zhifang Dong^2*^, Jun Liu^1^, Peter Axerio-Cilies^1^, Yehong Du^2^, Junjie Li^2^, Long Chen^2^, Lu Zhang^2^, Lidong Liu^1^, Jie Lu^1^, Chih-Hao Lu^3^, Yu-Jen Chang^3^, I-Hsien Teng^4,5^, Yi-Ru Chen^4,5^, Ning Zhou^6^, Dong Chuan Wu^4,5,7#^ and Yu Tian Wang^1#^

^1^DM Centre for Brain Health and Department of Medicine, Vancouver Coastal Health Research Institute, University of British Columbia, Vancouver, British Columbia V6T 2B5, Canada; ^2^Pediatric Research Institute, Ministry of Education Key Laboratory of Child Development and Disorders, National Clinical Research Center for Child Health and Disorders, China International Science and Technology Cooperation Base of Child Development and Critical Disorders, Chongqing Key Laboratory of Translational Medical Research in Cognitive Development and Learning and Memory Disorders, Children’s Hospital of Chongqing Medical University, Chongqing 400014, P. R. China; ^3^The Ph.D. Program of Biotechnology and Biomedical industry, College of Medicine, China Medical University, Taichung, Republic of China; ^4^Translational Medicine Research Center, China Medical University Hospital, Taichung, Republic of China, ^5^Graduate Institute of Biomedical Sciences, China Medical University, Taichung, Republic of China, ^6^iHuman Institute, ShanghaiTech University, Shanghai, P.R. China, ^7^Neuroscience and Brain Disease Center, China Medical University, Taichung, Republic of China.

*These authors contributed equally to this work

.

^#^Correspondence should be addressed to either DCW at [dongchuanwu@mail.cmu.edu.tw](mailto:dongchuanwu@mail.cmu.edu.tw) or YTW at [ytwang@brain.ubc.ca](mailto:ytwang@brain.ubc.ca)

**This PDF file includes:**

Figures. S1 to S8


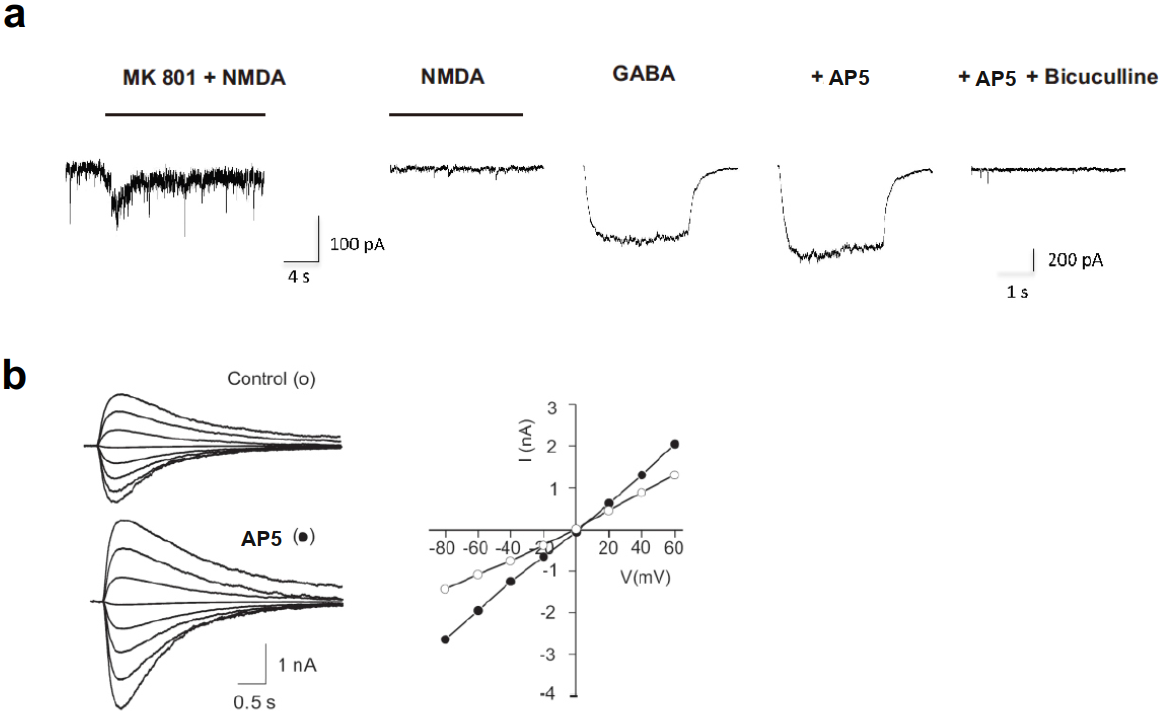


**Figure. S1**. AP5 potentiates GABA_A_R mediated currents via a NMDAR-independent mechanism in cultured hippocampal neurons. (**a**) AP5-induced potentiation did not require the opening of NMDAR-gated channels. Representative currents traces were taken under indicated recording conditions. Blockade of NMDA channels was produced by co-application of NMDA (50 μM) and MK-801 (10 μM) (MK801+NMDA) and evidenced by the in ability to produce a detectable current by a subsequent application of NMDA (50 μM; NMDA). Following NMDAR blockade, GABA (10 μM) currents induced in the presence of 200μM AP5 (+AP5) were much bigger than that in the absence of AP5 (GABA), and completely blocked by the addition of 10 μM bicuculline (+AP5+Bicuculline). (**b**) AP5 potentiation of GABA currents is not associated with alteration of the reversal potential. Left, representative current traces induced by pressure-ejection of GABA (10 μM) from a pipette at various holding potentials in the absence (Control) and presence of 200 µM AP5 (AP5). Right, current-voltage (*I-V*) relationship curves constructed from the currents shown on the Left showing that AP5 potentiation was not associated with an alteration of the reverse potential.


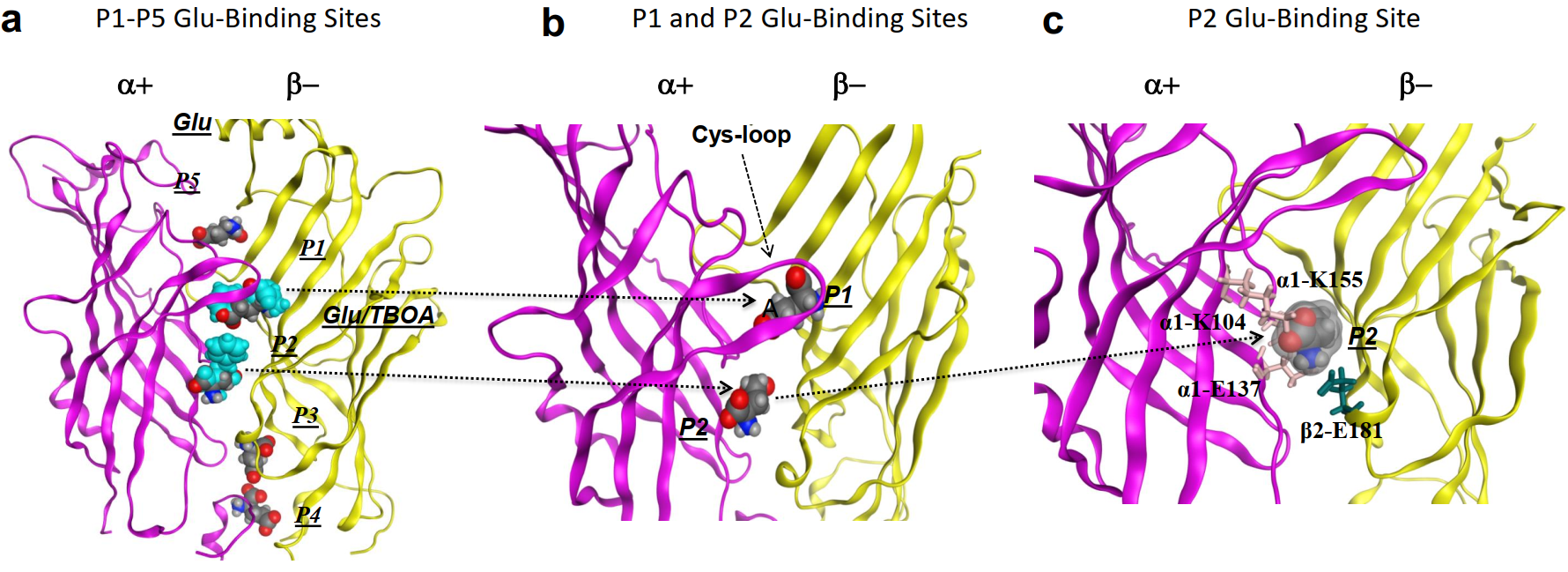


Figure. S2.

Simulated molecular dockings of glutamate onto the α+/β- interface of the modeled GABA_A_R. (**a**) Side perspective of the α+/β- interface predicts 5 putative glutamate (Glu) binding pockets (P1-P5). (**b**) Receptor modeling diagram enlarged from (**a**) further emphasizing on the two putative glutamate binding pockets (indicated in blue in **a**), one just behind the loop C (P2) and the other below the loop C (P2), that can accommodate both Glu and bigger glutamate-like molecule TBOA (Glu/TBOA). (**c**) Receptor structure modeling further enlarged from (**b**) illustrate the docked glutamate molecule and its interactions with critical residues in the identified glutamate-binding pocket at the α+/β- interface.


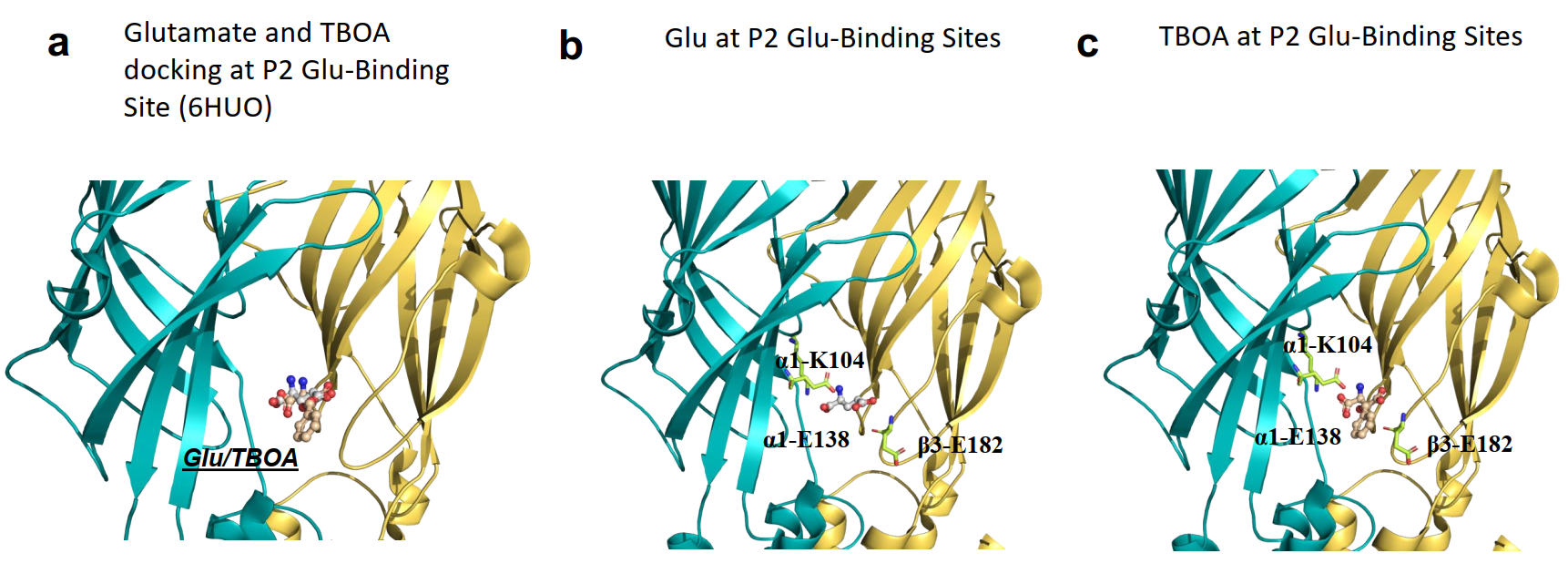


Figure. S3. Molecular dockings of glutamate onto the α^+^/β^-^ interface of the α1β3γ2 GABA_A_R (6HUO). (a) Side perspective of the α^+^/β^-^ interface of P2 that can accommodate both Glu and glutamate-like molecule TBOA (Glu/TBOA). (b) and (c) illustrate the docked glutamate or TBOA molecule and their interactions with critical residues in the P2 glutamate-binding pocket at the α+/β- interface.


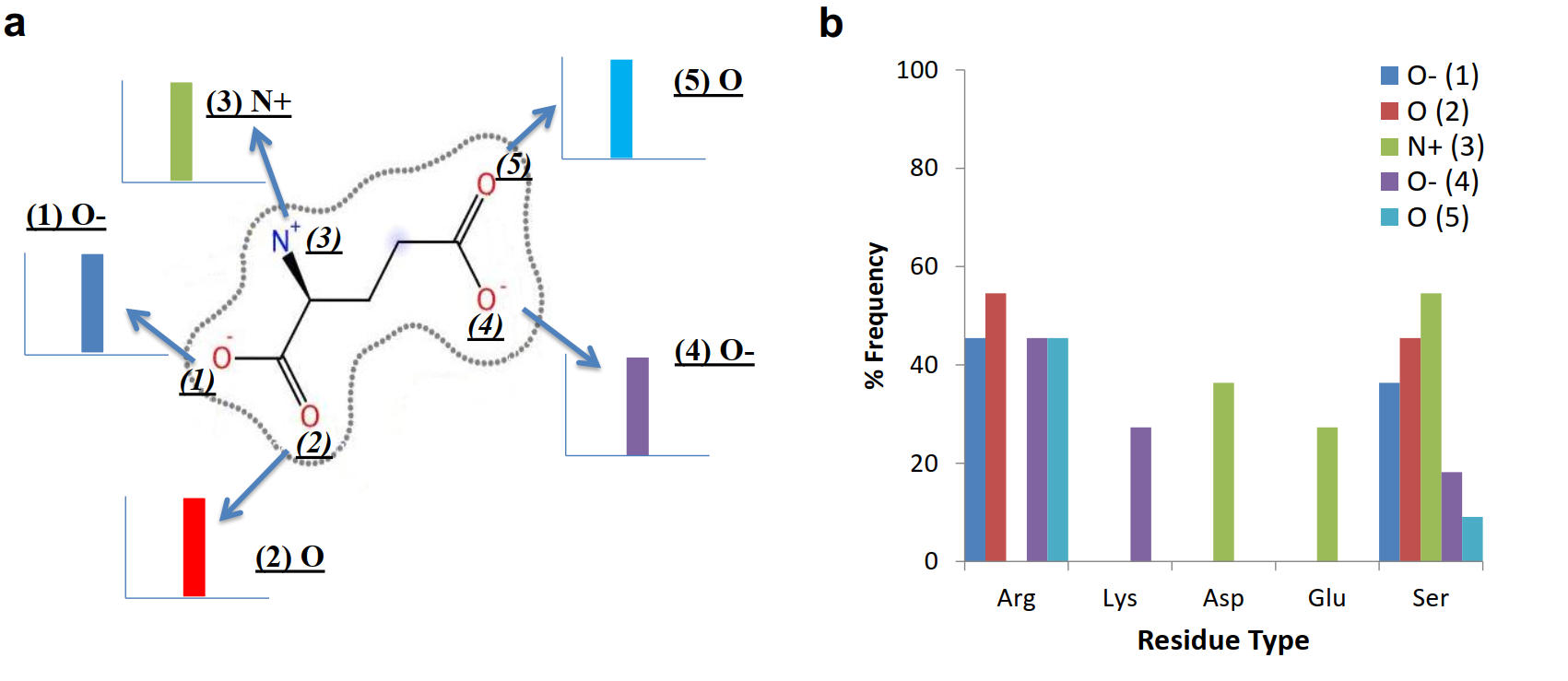


Figure. S4. Glutamate-binding proteins/receptors share van der Waals’ contacts formed between their common charged amino acid residues and glutamate molecule. Screening crystalized structures of glutamate-bound GluN2A[2A5S], GluN2D[3OEK/3OEL], GluA2[1FTJ], GluA4[3FAS], GluK2[1S7Y], mGluR1 1EWK], mGluR5[3LMK] and GluCl[3RIF] reveals the major types of the direct van der Waals’ contacts formed between glutamate and protein/receptor (a) and demonstrates that the most common pocket forming amino acid residues involved in direct interacting with glutamate are either positively or negatively charged residues Arg, Lys, Asp, Glu, and Ser (b). The panel b indicates the interaction frequency between these pocket forming residues and specific glutamate active groups.


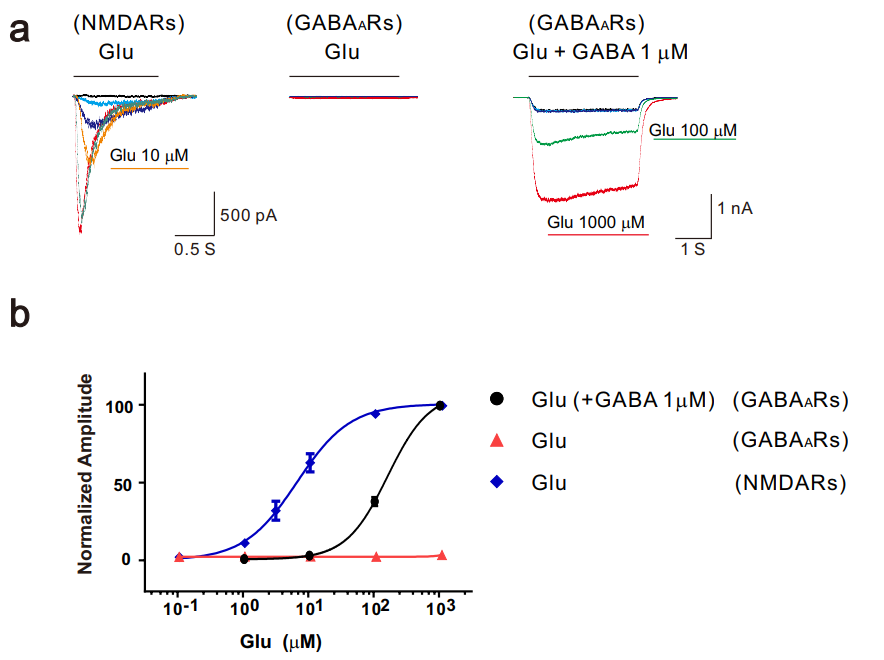


Figure. S5. Comparison of GABA and glutamate induced responses on GABA_A_R and NMDAR (GluN1 and GluN2A). (a) Representative current traces of glutamate induced dose responses on NMDARs (N=5), dose responses of glutamate potentiation of GABA induced currents on GABA_A_Rs (N=14) and glutamate induced dose responses on GABA_A_Rs (N=3). (b) The dose-response curves for NMDARs and GABA_A_Rs normalized to maximal currents induced by glutamate or glutamate with GABA. The dose-response curves for GABA_A_Rs induced by glutamate normalized to the currents induced by 1 mM GABA.


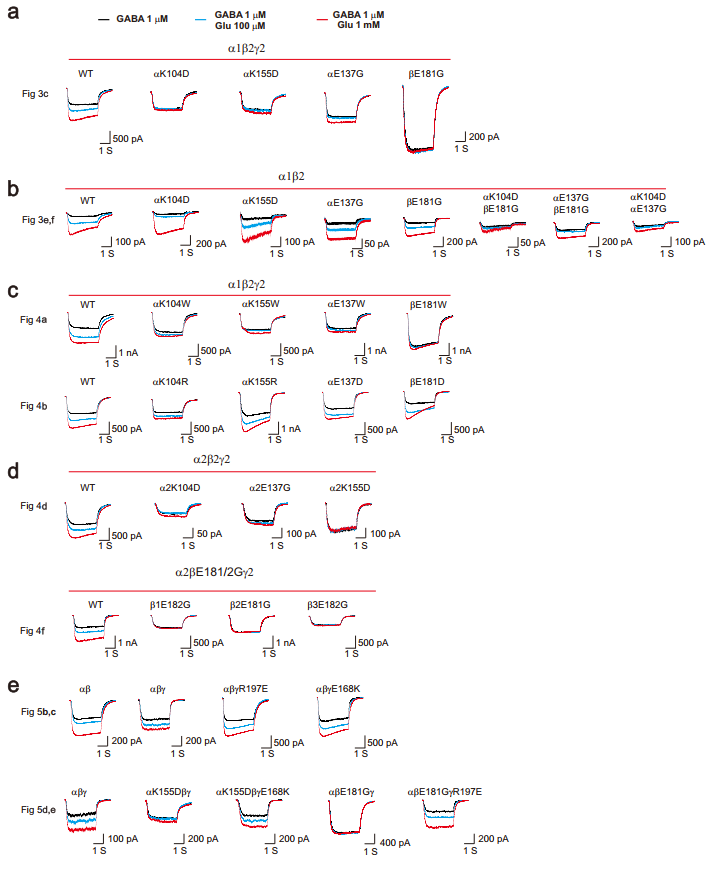


Figure. S6. Representative current traces of glutamate potentiated GABA responses on different mutations of GABA_A_Rs. Representative current traces of glutamate induced changes of GABA responses from main Fig. 3c (a), Fig. 3e (b), Fig. 4a and b (c), Fig 4d and f (d) and Fig. 5b, c, d and e (e), respectively.


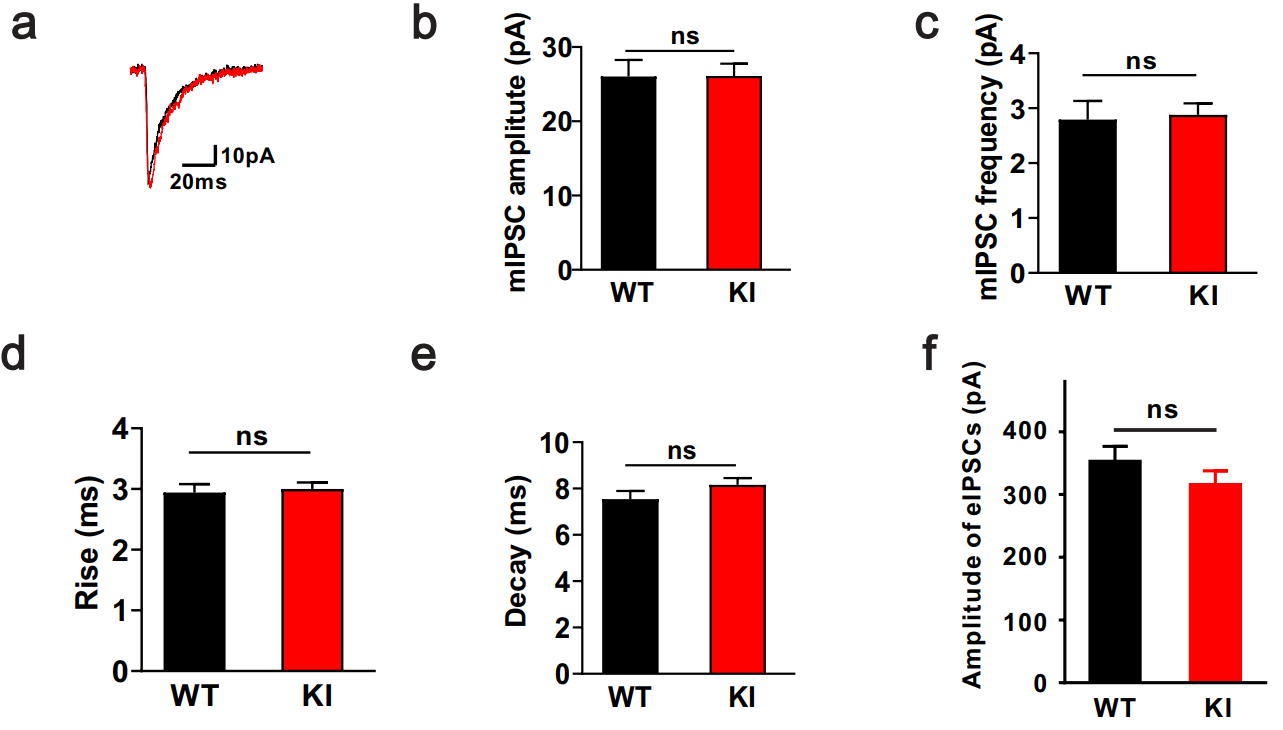


Figure. S7. β2_E181G_ knock-in (KI) mice did not affect basic properties of inhibitory synaptic transmission. (a) Representative average mIPSC from CA1 pyramidal neurons in WT and KI mice. The amplitude (b) and frequency (c) of mIPSCs in KI mice remained unchanged compared with WT (n=21 for WT; n=23 for KI). The mean rise time (d) and decay time constant (e) of mIPSCs did not show the significant differences between WT and KI mice (n=21 for WT; n=23 for KI). (f) The amplitude eIPSCs in KI mice remained unchanged compared with WT (n=42 for WT; n=35 for KI).


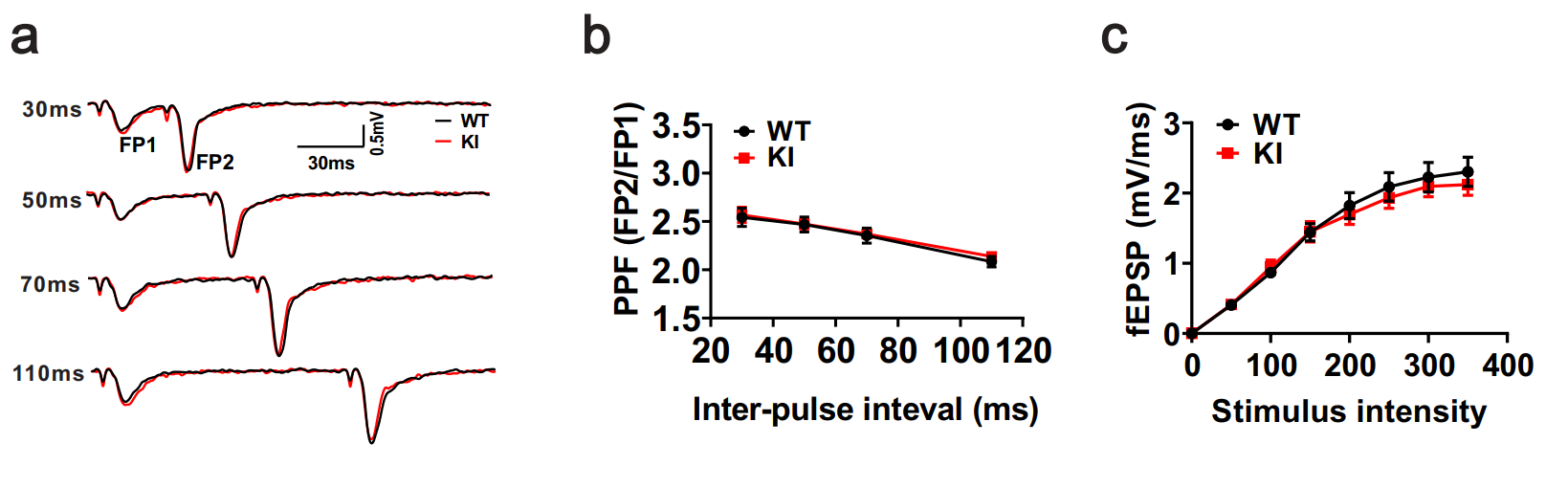


Figure. S8. β2_E181G_ knock-in (KI) mice did not affect presynaptic release. (a) Representative paired-pulse facilitation (PPF) trace at 30, 50, 70 and 110 ms inter-pulse intervals, respectively. There were no significant differences between WT and KI mice (n=32 slices for WT; n=34 slices for KI) (b). The input-output relationship did not change in KI mice compared with WT (c).
